# The quantity of CD40 signaling determines the differentiation of B cells into functionally distinct memory cell subsets

**DOI:** 10.7554/eLife.44245

**Published:** 2019-06-21

**Authors:** Takuya Koike, Koshi Harada, Shu Horiuchi, Daisuke Kitamura

**Affiliations:** Division of Molecular Biology, Research Institute for Biomedical Sciences (RIBS)Tokyo University of ScienceNodaJapan; Ragon Institute of MGH, MIT and HarvardUnited States; Institute of Industrial Science, The University of TokyoJapan

**Keywords:** memory B cells, germinal center, plasma cells, immune response, recall response, Mouse

## Abstract

In mice, memory B (B_mem_) cells can be divided into two subpopulations: CD80^hi^ B_mem_ cells, which preferentially differentiate into plasma cells; and CD80^lo^ B_mem_ cells, which become germinal center (GC) B cells during a recall response. We demonstrate that these distinct responses can be B-cell-intrinsic and essentially independent of B-cell receptor (BCR) isotypes. Furthermore, we find that the development of CD80^hi^ B_mem_ cells in the primary immune response requires follicular helper T cells, a relatively strong CD40 signal and a high-affinity BCR on B cells, whereas the development of CD80^lo^ B_mem_ cells does not. Quantitative differences in CD40 stimulation were enough to recapitulate the distinct B cell fate decisions in an in vitro culture system. The quantity of CD40 signaling appears to be translated into NF-κB activation, followed by BATF upregulation that promotes B_mem_ cell differentiation from GC B cells.

## Introduction

Memory B (B_mem_) cells are crucial for humoral immunity, preventing the spread of re-infecting viruses and bacteria by rapidly producing large amounts of class-switched antibodies (Abs) against these pathogens. B_mem_ cells also regenerate themselves with improved affinity for their cognate antigen through the germinal center (GC) reaction upon each iterative infection. The rapid and exaggerated response of B_mem_ cells has been attributed to their B-cell receptor (BCR) isotype, namely membrane-bound IgG (mIgG). The relatively long cytoplasmic tail of mIgG, as compared to that of membrane-bound IgM (mIgM) which consists of only three amino acids, contains specific motifs that recruit signaling molecules such as Grb2 and SAP97 after BCR cross-linking; these motifs and molecules are required for enhanced BCR signaling and plasma cell (PC) formation upon rechallenge (Engels et al., 2009; Kaisho et al., 1997; Liu et al., 2012; Lutz et al., 2015). At odds with this model is the finding that naïve B cells expressing NP-specific mIgG1, which were derived from cloned mice generated from a single IgG1^+^ B_mem_ cell, expanded to a similar extent as NP-specific (IgH-knock-in) IgM^+^ naïve B cells, and both B cell types predominantly differentiated into GC B cells rather than PCs upon primary immunization (Kometani et al., 2013). These observations suggest that the heightened capacity of B_mem_ cells to differentiate into PCs cannot be attributed solely to the expression of mIgG1, but also depends on some cell-intrinsic status, such as a reduced expression of Bach2, a transcription factor that suppresses PC differentiation (He et al., 2017; Kometani et al., 2013). In support of this notion, human B_mem_ cells differentiate into PCs better than do naïve B cells in antigen-free in vitro cultures (Arpin et al., 1997).

As a further refinement in our understanding of the functional properties of B_mem_ cells, it has recently been proposed that the B_mem_ cell pool can be divided into distinct subsets on the basis of their potential to generate PCs or GC B cells upon antigen encounter. Earlier reports indicated that B_mem_ cells consist of mIgG^+^ cells and mIgM^+^ cells, with the former prone to becoming PCs and the latter GC B cells (Dogan et al., 2009; Pape et al., 2011), although a recent report suggests a more complex scenario (McHeyzer-Williams et al., 2015). Another study proposed that functionally different subsets can be phenotypically defined using surface markers, CD80 and PD-L2: CD80^+^ PD-L2^+^ B_mem_ cells are prone to differentiate into PCs, whereas CD80^−^ PD-L2^−^ B_mem_ cells preferentially form GC upon secondary immunization, and CD80^−^ PD-L2^+^ B_mem_ cells are intermediate between the two but functionally closer to the CD80^−^ PD-L2^−^ B_mem_ cells (Zuccarino-Catania et al., 2014). Consistent with the BCR-isotype-based classification, the majority of IgG1^+^ B_mem_ cells had the CD80^+^ PD-L2^+^ phenotype, whereas IgM^+^ B_mem_ cells were dominated by CD80^−^ PD-L2^−^ cells. In addition, CD73 has been used to further dissect the PC-prone subpopulation as CD80^+^ PD-L2^+^ CD73^+^ B_mem_ cells (He et al., 2017).

Circulating memory T cells have also been functionally divided into two subsets termed effector memory T (T_EM_) and central memory T (T_CM_) cells. T_EM_ cells, which lack the lymph node homing receptors CD62L and CCR7, produce abundant effector cytokines and cytotoxic granules, whereas T_CM_ cells expressing both CD62L and CCR7 have a greater potential for proliferation and self-renewal (Mueller et al., 2013). The PC-prone B_mem_ cells may resemble the T_EM_ cells in terms of their effector function, whereas the GC-B cell-prone B_mem_ cells are similar to the self-renewable T_CM_ cells.

At present, it is not clear how these two subsets diverge during the primary immune response. It has been reported that CD80^+^ PD-L2^+^ B_mem_ cells have the highest affinity for antigen, whereas the double negative cells have the lowest affinity (Tomayko et al., 2010; Zuccarino-Catania et al., 2014). Moreover, it is generally accepted that B cells expressing BCR of higher affinity are prone to differentiate into PCs and those of lower affinity into GC B cells (Ochiai et al., 2013; Paus et al., 2006; Sciammas et al., 2011). Together with the analysis of the frequency of BCR somatic mutations and the timing of B_mem_ cell generation (Weisel et al., 2016), it has been suggested that PC-prone B_mem_ cells, including IgG1^+^ and CD80^+^ PD-L2^+^ B_mem_ cells, are mainly derived from the GC, whereas most of GC-B cell-prone B_mem_ cells, including IgM^+^ and CD80^−^ PD-L2^−^ B_mem_ cells, are generated before GC formation. It remains unclear, however, which signals determine the distinct B_mem_ cell fates. Thus, we sought to define the signaling mechanism for the bifurcated generation of B_mem_ cells.

## Results

### Characterization of CD80^hi^ and CD80^lo^ memory B cells

**Figure 1.**
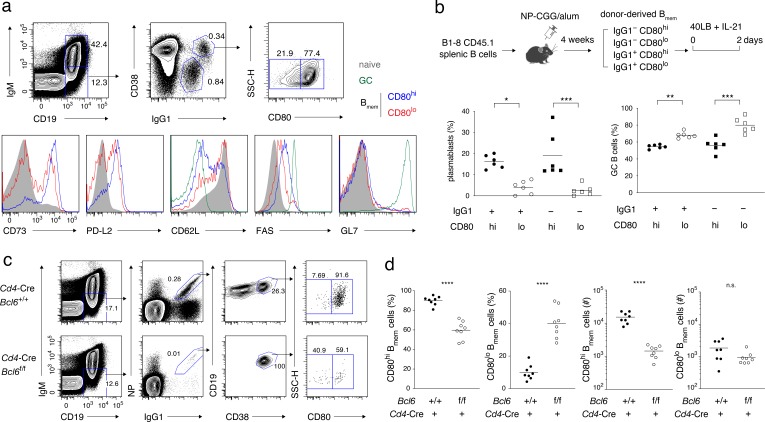
Characterization of CD80^hi^ and CD80^lo^ memory B cells. (**a**) Splenocytes from B6 mice immunized with NP-CGG in alum 4 weeks earlier were analyzed by flow cytometry (FCM). The gating strategy for naïve B cells (CD19^+^ IgM^+^), GC B cells (CD19^+^ IgM^−^ IgG1^+^ CD38^−^), and CD80^lo^ and CD80^hi^ B_mem_ cells (CD19^+^ IgM^−^ IgG1^+^ CD38^+^) is shown in the top panels, and the expression of the indicated cell-surface proteins in each population is shown in the bottom panels. Data are representative of two independent experiments with similar results. (**b**) Outline of the experimental protocol (top). Splenic B cells from CD45.1 B1-8 ki mice were transferred into B6 mice (CD45.2), which were then immunized with NP-CGG in alum. Four weeks later, four subsets of donor-derived B_mem_ cells (CD45.1^+^ CD19^+^ CD38^+^), defined by the expression of IgG1 and CD80, were sorted from recipient spleens, cultured on 40LB feeder layers with IL-21 for 2 days, and analyzed by FCM. The frequency of CD138^+^ GL7^−^ plasmablasts or PCs and CD138^−^ GL7^+^ GC B cells in each subset is represented by a dot (bottom; combined data from two triplicate experiments). (**c**) Splenocytes from *Cd4-*Cre, *Bcl6*^+/+^ or *Bcl6*^f/f^ mice immunized with NP-CGG in alum 6 weeks earlier were analyzed by FCM. The representative data indicate the gating strategy with percentages of the gated population. (**d**) The frequency (%) and absolute number (#) of CD80^hi^ and CD80^lo^ cells among IgG1^+^ B_mem_ cells in each spleen from individual recipient mice, as analyzed in (**c**) (n = 8). ﻿The mean of the values in each group is indicated by a horizontal bar (**b, d**). n.s., not significant (p>0.05); *, p<0.05; ***, p<0.001; ****, p<0.0001; as determined by one-way ANOVA followed by Tukey’s multiple comparisons test (**b**) and unpaired Student’s *t* test (**d**). All data are representative of two independent experiments, except (**b** and **d**), where data from two independent experiments are combined. 10.7554/eLife.44245.004Figure 1—source data 1.Source data for Figure 1b and d.

On the basis of previous reports showing that IgG1^+^ B_mem_ cells are mainly composed of CD80^+^ PD-L2^+^ and CD80^–^ PD-L2^+^ B_mem_ cells and that CD80^–^ PD-L2^+^ and CD80^–^ PD-L2^–^ B_mem_ cells are functionally similar (He et al., 2017; Zuccarino-Catania et al., 2014), we hypothesized that the proposed B_mem_ cell subsets could be distinguished simply by the expression of CD80, as CD80^hi^ or CD80^lo^ B_mem_ cells. Consistent with these reports, most of the CD80^hi^ B_mem_ cells expressed PD-L2 and CD73, thus constituting the reported affinity-matured subset (He et al., 2017; Tomayko et al., 2010; Zuccarino-Catania et al., 2014), whereas the CD80^lo^ B_mem_ cells consisted of PD-L2^+^ and PD-L2^−^, as well as CD73^+^ and CD73^−^ subpopulations (Figure 1a). Although CD62L was reported to be expressed on B_mem_ cells (Anderson et al., 2007), the majority of CD80^hi^ B_mem_ cells express a lower level of CD62L (a phenotype that somewhat resembles T_EM_ cells), whereas most CD80^lo^ B_mem_ cells express a higher level of CD62L (a phenotype that somewhat resembles T_CM _cells). Both CD80^hi^ and CD80^lo^ B_mem_ cells expressed FAS (also called CD95 or APO1) at a higher level than naïve B cells, as previously reported (Anderson et al., 2007), but at a lower level than GC B cells, and, as expected, they were GL7^–^.

**Figure 1—figure supplement 1.**
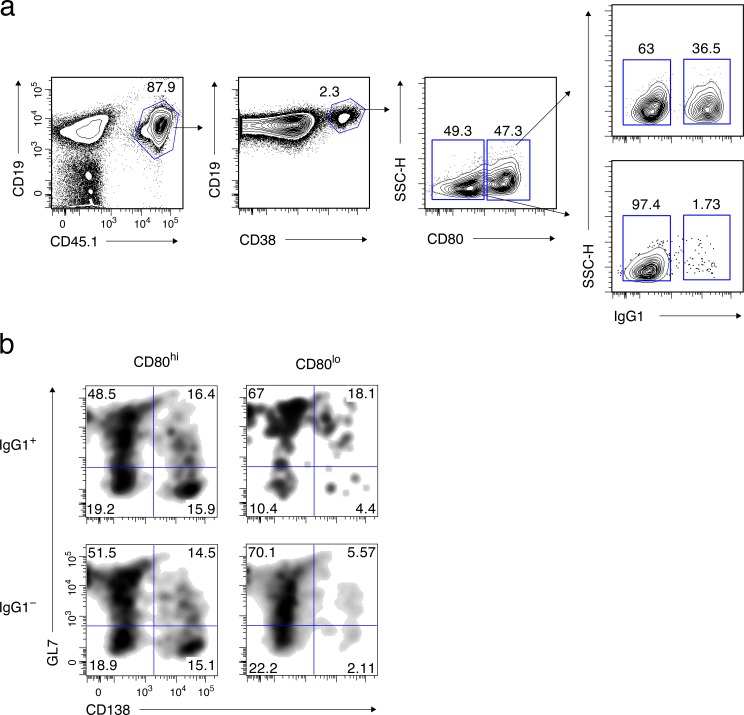
Supplementary data for Figure 1. (**a**) Sorting strategy for Figure 1b. Splenic B cell from CD45.1 B1-8 ki mice were transferred into B6 mice (CD45.2), which were then immunized with NP-CGG in alum. Four weeks later, donor-derived cells were enriched from pooled splenocytes by magnetic sorting, and further sorted into four B_mem_ cell subsets, as described in the text. (**b**) Gating strategy for Figure 1b. Four B_mem_ subsets, sorted as above, were cultured on 40LB feeder layers with IL-21 for 2 days, and analyzed by FCM. Feeder cells were gated out as CD45.1^–^cells. The expression of GL7 and CD138 in CD45.1^+^ cells is shown.

In order to examine in vitro whether the CD80^hi^ and CD80^lo^ B_mem_ cells are intrinsically biased in their differentiation fate toward PCs or GC B cells, we transferred into B6 mice allotypically marked (CD45.1^+^) B cells of B1-8 knock-in (ki) mice, whose knock-in IgH chain, when combined with the λL chain, forms an NP-specific BCR, and immunized these mice with NP-CGG. From these mice, we sorted CD80^hi^ and CD80^lo^ B_mem_ cells, either IgG1^+^ or IgG1^−^, and cultured them with IL-21 on feeder cells that express exogenous CD40L and BAFF (40LB) (Nojima et al., 2011; Takatsuka et al., 2018). Under these conditions, CD80^hi^ B_mem_ cells differentiated more preferentially into CD138^+^ plasmablasts or PCs and less into GL7^+^ GC-like B cells, as compared with CD80^lo^ B_mem_ cells, regardless of their BCR isotype (Figure 1b and Figure 1—figure supplement 1a,b). These in vitro data were consistent with the previous in vivo data (Zuccarino-Catania et al., 2014), and further revealed that the biased differentiation of the CD80^hi^ or CD80^lo^ B_mem_ cells is determined in a cell-intrinsic manner, and is essentially independent of BCR isotype and BCR affinity for antigen.

### Strong CD40 signaling induced by T_FH_ cells is required for the development of CD80^hi^ B_mem_ cells

We next sought to clarify a need for GC in the development of CD80^hi^ and CD80^lo^ B_mem_ cells. A previous report indicated that CD80 and PD-L2 were expressed at normal levels on B_mem_ cells in B-cell-specific BCL6-deficient mice that lack GCs (Kaji et al., 2012). To examine a role for the GC environment in B_mem_ cell development from normal B cells, we used CD4^+^ T-cell-specific BCL6-deficient mice, which lack T_FH_ cells and GCs (Kaji et al., 2012). Six weeks after immunization, the number of CD80^hi^ B_mem_ cells decreased by approximately ten-fold in *Cd4*-Cre *Bcl6*^f/f^ mice as compared to the control *Cd4*-Cre *Bcl6*^+/+^ mice, while the number of CD80^lo^ B_mem_ cells was essentially unchanged (Figure 1c,d). These data suggested that the GC environment, or more specifically T_FH_ cells, facilitate the development of CD80^hi^ B_mem_ cells.

**Figure 2.**
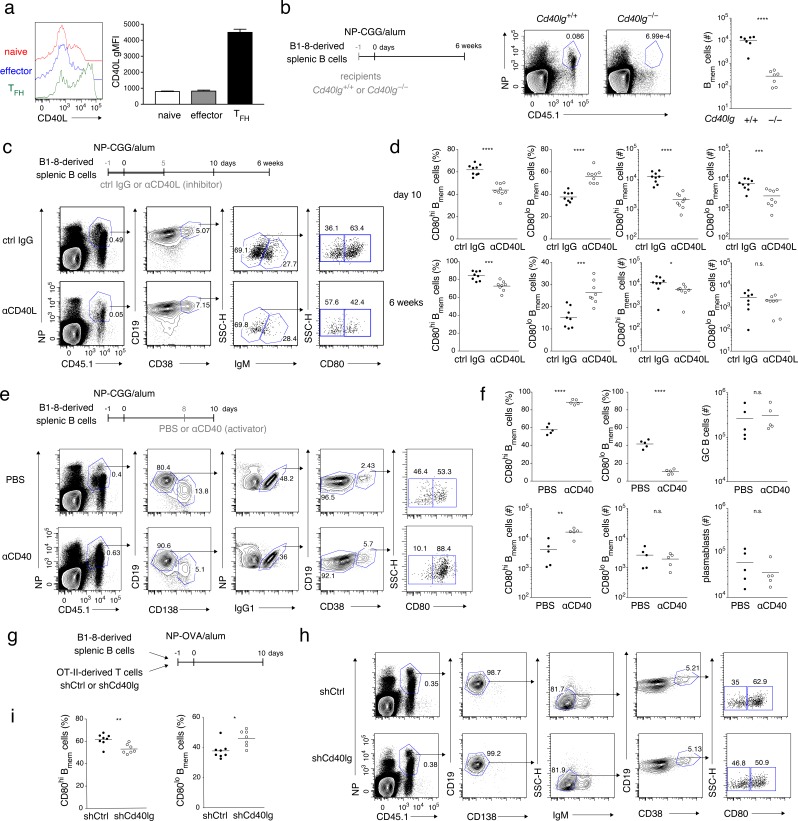
Strong CD40 signaling favors CD80^hi^ B_mem_ cell development. (**a**) Naïve T (CD4^+^ CD62L^+^ CXCR5^−^ PD-1^−^), effector T (CD4^+^ CD62L^−^ CXCR5^−^ PD-1^−^), and T_FH_ (CD4^+^ CD62L^−^ CXCR5^+^ PD-1^+^) cells were sorted from spleens of mice immunized with NP-CGG in alum 7 days earlier and then stimulated with phorbol myristate acetate (PMA) and ionomycin for 2 hr. CD40L expression on each cell subset was analyzed by FCM (left) and represented as geometric mean fluorescence intensity (gMFI, right) (mean + s.d. of triplicates). (**b**) *Cd40lg*^+/+^ or *Cd40lg*^−/−^ mice were transferred with splenic B cells from B1-8 ki mice, and immunized with NP-CGG in alum. Six weeks later, the frequency of donor-derived (CD45.1^+^) NP^+^ CD19^+^ B cells (representative data on the left) and the number of the donor-derived B_mem_ cells (CD19^+^ CD45.1^+^ NP^+^ CD38^+^; plotted on the right; n = 7) in each spleen were analyzed by FCM. (**c, d**) B6 mice were transferred with splenic B cells from B1-8 ki mice, immunized with NP-CGG in alum, and injected subcutaneously (s.c.) with an inhibitory CD40L (MR-1: 1 mg/kg) mAb (αCD40L) or an isotype-matched control (ctrl IgG) Ab every day from day −1 to day 5 after immunization. Ten days or 6 weeks after immunization, splenocytes of the recipient mice were analyzed by FCM. (**c**) Representative data (day 10) of the analysis showing the gating strategy. (**d**) The frequency (%) of CD80^hi^ and CD80^lo^ cells in the donor-derived, class-switched B_mem_ cells (CD45.1^+^ NP^+^ CD19^+^ CD38^+^ IgM^−^), and their absolute numbers (#) at 10 days (top, n = 9) and 6 weeks (bottom, n = 8) after immunization are plotted. (**e, f**) B6 mice transferred with B1-8 ki B cells and immunized as in (**c, d**) were injected intraperitoneally (i.p.) with PBS or a stimulatory CD40 mAb (αCD40) (FGK4.5: 250 μg) at 8 days after immunization. Ten days after immunization, splenocytes from the recipient mice were analyzed by FCM. (**e**) Representative data of the analysis showing the gating strategy. (**f**) The frequency (%) and absolute numbers (#) of CD80^hi^ and CD80^lo^ cells in the donor-derived, IgG1^+^ B_mem_ cells (CD45.1^+^ NP^+^ CD138^–^ CD19^+^ IgG1^+^ CD38^+^), and the numbers of the donor-derived GC B cells (CD45.1^+^ NP^+^ CD19^+^ IgG1^+^ CD38^−^) or of plasmablasts (CD45.1^+^ NP^+^ CD138^+^) in splenocytes are plotted (n = 5). (**g–i**) B6 mice, co-transferred on day −1 with B1-8 ki B cells (1 × 10^5^) and OT-II T cells (1 × 10^5^) that had been transduced with control (shCtrl) or shCd40lg retroviral vectors on the previous day, were immunized with NP-OVA in alum. Ten days after immunization, spleen cells from the recipient mice were analyzed by FCM. (**g**) Outline of the experimental protocol. (**h**) Representative data showing the gating strategy. (**i**) The frequencies of CD80^hi^ and CD80^lo^ cells among the donor-derived, class-switched B_mem_ cells, defined as in (**d**) (n = 8). The mean of the values in each group is indicated by a horizontal bar (**b, d, f, i**). n.s., not significant (p>0.05); *, p<0.05; **, p<0.01; ***, p<0.001; ****, p<0.0001; unpaired Student’s *t* test (**b, d, f, i**). All data are representative of two independent experiments except (**b**) and (**i**), where data from two independent experiments are combined. 10.7554/eLife.44245.007Figure 2—source data 1.Source data for Figure 2b, d, f and i.

T_FH_ cells differ from naïve or effecter CD4^+^ T cells in that they express a much higher level of CD40L (Breitfeld et al., 2000), as we confirmed using purified T-cell subsets (Figure 2a and Figure 2—figure supplement 1a). As previously indicated (Lenschow et al., 1994; Ranheim and Kipps, 1993), stimulation of B cells through CD40, but not BCR, induced CD80 expression in a time- and dose-dependent manner (Figure 2—figure supplement 1b,c). Supposing that CD80 expression on B cells that is induced during the primary response is maintained until the B_mem_ cell stage, we hypothesized that T_FH_ cell stimulation through CD40 promotes CD80^hi^ B_mem_ cell development. To test this, we first used CD40L-deficient mice as recipients of antigen-specific (BCR-knock-in) naïve B cells. After immunization of such mice, however, B_mem_ cell development from the donor B cells was abrogated altogether in the absence of CD40L, indicating that CD40L-mediated stimulation is indispensable for B_mem_ cell development (Figure 2b).

**Figure 2—figure supplement 1.**
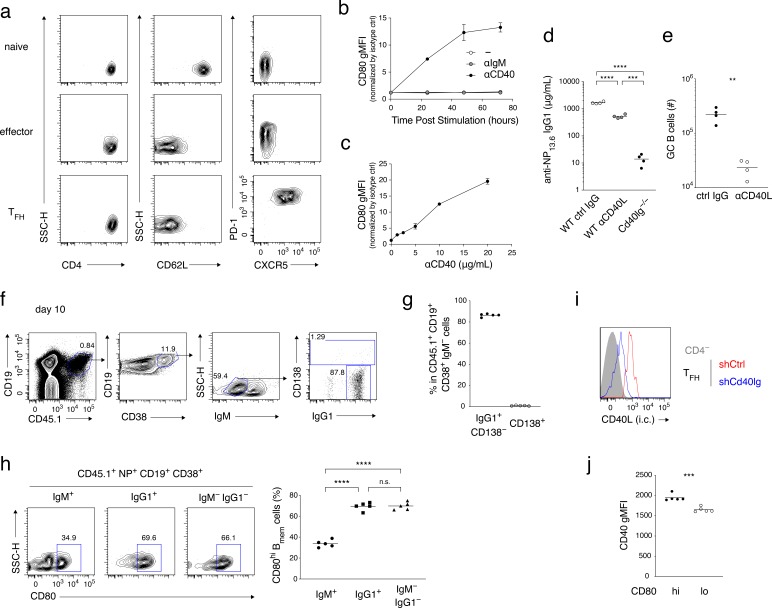
Supplementary data for Figure 2. (**a**) Naïve T (CD4^+^ CD62L^+^ CXCR5^−^ PD-1^−^), effector T (CD4^+^ CD62L^−^ CXCR5^−^ PD-1^dull^), and T_FH_ (CD4^+^ CD62L^−^ CXCR5^+^ PD-1^+^) cells were sorted from spleen of mice immunized 10 days previously, for the experiments whose results are summarized in Figure 2a. The FCM profiles that are shown demonstrate the purity of the sorted fractions. (**b**) Naïve B cells were cultured with anti-CD40 Ab (10 μg/ml), anti-IgM Ab (10 μg/ml), or medium alone, for the indicated length of time, then stained with anti-CD80 antibody, and analyzed by FCM. gMFI of the CD80 staining was normalized to that of staining with isotype-matched control Ab. Mean ± s.d. of triplicates. (**c**) Naïve B cells were cultured with the indicated concentration of anti-CD40 Ab for 3 days, and analyzed as in (**b**). Mean ± s.d. of triplicates. (**d**) The concentration of anti-NP_13.6_ IgG1 Ab in the sera at 10 days after the immunization of the mice shown in Figure 2c,d and Figure 2b (*Cd40lg*^−/−^ recipients) (n = 4). (**e**) Absolute number (#) of GC B cells (CD45.1^+^ NP^+^ CD19^+^ CD38^−^) in the spleens at 10 days after immunization of the mice shown in Figure 2c,d (n = 4). (**f–h**) Splenic B cells from CD45.1 B1-8 ki mice were transferred into B6 mice (CD45.2), which were then immunized with NP-CGG in alum. Splenocytes from the recipient mice at day 10 after immunization were analyzed by FCM. (**f**) Gating strategy for (**g**). (**g**) The frequency (%) of IgG1^+^ CD138^−^ and CD138^+^ cells in CD45.1^+^ CD19^+^ CD38^+^ IgM^−^ cells (n = 5). (**h**) Representative FCM data showing the expression of CD80 (left) and the frequency (%) of CD80^hi^ cells (right; n = 5) in B_mem_ cells (CD45.1^+^ NP^+^ CD19^+^ CD38^+^) of the indicated isotype. (**i**) Knock-down efficiency of shCd40lg vector in T_FH_ cells. Intracellular (i.c.) CD40L expression in shCtrl- or shCd40lg-transduced T_FH_ cells and CD4^−^ cells in the mice described in Figure 2g–i was analyzed by FCM. (**j**) CD40 expression on CD80^hi^ and CD80^lo^ B_mem_ cells (CD45.1^+^ NP^+^ CD19^+^ CD38^+^ IgM^−^) was analyzed by FCM at day 10 after immunization. gMFI of the CD40 staining is plotted (n = 5).

Next, we treated immunized mice with anti-CD40L blocking antibody (Ab) in a dose that we had determined only partially inhibited Ab production and GC B-cell formation (Figure 2—figure supplement 1d,e). This treatment preferentially affected CD80^hi^ B_mem_ cell development. At 10 days after immunization, the frequency of CD80^hi^ B_mem_ cells was significantly reduced among class-switched B_mem_ cells, of which ~90% were IgG1^+^ and ~10% were IgM^–^ IgG1^–^, both normally containing the CD80^hi^ cells at a similar frequency (Figure 2c,d and Figure 2—figure supplement 1f–h). The absolute number of the CD80^hi^ B_mem_ cells was also severely reduced while there was only a moderate reduction in the absolute number of CD80^lo^ B_mem_ cells, in a condition where the number of GC B cells was reduced by about ten-fold (Figure 2d and Figure 2—figure supplement 1e). The frequency and the number of the CD80^hi^ B_mem_ cells were somewhat recovered by 6 weeks after immunization, presumably because of generation of such cells in the late GC after the lapse of the injected anti-CD40L Ab (Figure 2d). In order to focus on B_mem_ cell generation, avoiding possible effects of alteration in GC formation or B_mem_ cell maintenance, we hereafter mainly analyzed B_mem_ cells by 10 days after immunization, referring to previous reports (Suan et al., 2017; Wang et al., 2017; Weisel et al., 2016). As an opposite experiment, administration of agonistic anti-CD40 Ab to immunized mice markedly increased the frequency and the number of CD80^hi^ B_mem_ cells, while the numbers of CD80^lo^ B_mem_ cells, GC B cells and plasmablasts remained unchanged (Figure 2e,f).

Finally, antigen (NP)-specific B cells and carrier [ovalbumin (OVA)]-specific (OT-II) CD4^+^ T cells, which had been transduced with short hairpin (sh) RNA targeting CD40L (shCd40lg) or unrelated control (shCtrl), were co-transferred into B6 mice, which were then immunized with NP-OVA (Figure 2g). Among the generated donor-derived class-switched B_mem_ cells, the frequency of CD80^hi^ B_mem_ cells was significantly lower in mice that had received the CD40L-knockdown T cells as compared to those that had received the control T cells (Figure 2g–i and Figure 2—figure supplement 1i). These data together suggest that, although CD40L-mediated stimulation is required for development of both B_mem_ cell types, stronger CD40 stimulation by T_FH_ cells selectively facilitates the development of CD80^hi^ B_mem_ cells.

### CD40 signal strength in vitro affects differentiation into CD80^hi^ or CD80^lo^ B_mem_ cells in vivo

**Figure 3.**
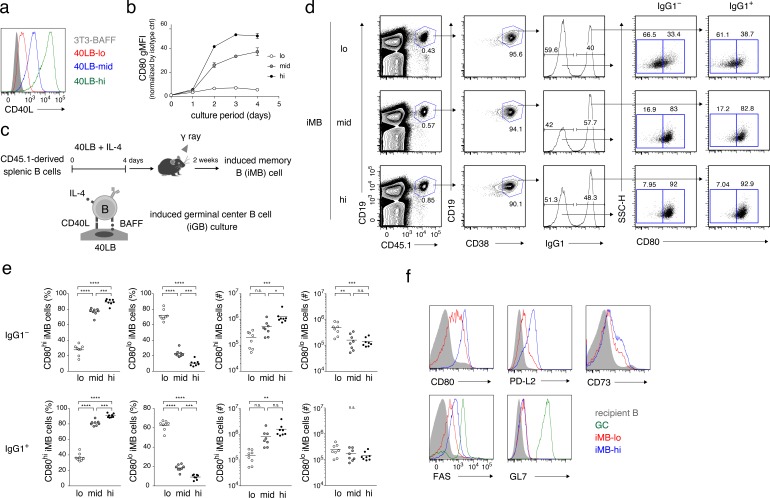
The quantity of CD40 signaling determines the differentiation of B cells into distinct B_mem_ cell subsets. (**a**) Expression of CD40L on 3T3-BAFF cells and 40LB sublines (40LB-lo, 40LB-mid, and 40LB-hi) was analyzed by FCM. (**b**) Splenic B cells were cultured with IL-4 for the indicated number of days on feeder layers of each 40LB subline. The expression of CD80 on the expanded B (iGB) cells was analyzed by FCM and presented as gMFI (mean of triplicates). (**c**) A schematic representation of a method used to generate the induced memory B (iMB) cells. Splenic B cells from CD45.1^+^ congenic B6 mice were cultured for 4 days, as in (**b**). The resultant iGB-lo, iGB-mid, or iGB-hi cells were transferred intravenously (i.v.) into γ-irradiated mice (CD45.2^+^), and the donor-derived B_mem_-like cells (CD19^+^ CD45.1^+^ CD38^+^) detected in the recipient spleens 2 weeks after the transfer were designated iMB-lo, iMB-mid or iMB-hi cells, respectively. (**d–f**) Expression of CD80 on IgG1^+^ or IgG1^–^ iMB cells (iMB-lo, iMB-mid or iMB-hi) generated as in (**c**) was analyzed by FCM. (**d**) Representative data showing the gating strategy. (**e**) The frequencies of CD80^hi^ and CD80^lo^ cells among the IgG1^+^ or IgG1^–^ iMB cells (%) and their absolute number per spleen (#) are plotted (n = 8). (**f**) Expression of the indicated surface markers on the recipient total B cells (CD45.1^−^ CD19^+^), spontaneous GC B cells (CD45.1^−^ CD19^+^ CD38^−^ GL7^+^), the iMB-lo and the iMB-hi cells (CD19^+^ CD45.1^+^ CD38^+^). The mean of the values in each group is indicated by a horizontal bar (**e**). n.s., not significant (p>0.05); *, p<0.05; **, p<0.01; ***, p<0.001; ****, p<0.0001; as determined by one-way ANOVA followed by Tukey’s multiple comparisons test (**e**). All data are representative of two independent experiments except (**e**), where data from two independent experiments are combined. 10.7554/eLife.44245.010Figure 3—source data 1.Source data for Figure 3b and e.

The data discussed above strongly suggested that quantitative differences in CD40 signaling in B cells during the primary response determine their developmental fate into either CD80^hi^ or CD80^lo^ B_mem_ cells. However, these in vivo experiments cannot exclude the possibility that some other factors that are affected by the CD40/CD40L manipulation might contribute to the fate decision. In order to demonstrate a direct contribution of CD40 signaling quantity in B cells, we utilized our in vitro induced GC B (iGB) cell culture system, in which naïve B cells massively proliferate, efficiently switch to IgG1, and differentiate into GC-like B (iGB) cells after being cultured with IL-4 on a feeder layer of 40LB cells (Nojima et al., 2011). In addition, these iGB cells differentiate into memory-like B cells [termed induced memory B (iMB) cells] in vivo when transferred into irradiated mice (Nojima et al., 2011). To stimulate B cells through CD40 at different levels in this in vitro system, we derived 40LB sublines that express CD40L at low, intermediate and high levels, termed 40LB-lo, 40LB-mid, and 40LB-hi, respectively (Figure 3a). As expected, B cells cultured on 40LB-hi (iGB-hi cells) expressed the highest level of CD80, whereas those on 40LB-mid (iGB-mid cells) or 40LB-lo (iGB-lo cells) exhibited intermediate or the lowest levels of CD80 expression, respectively (Figure 3b). The iGB-hi, iGB-mid and iGB-lo cells underwent class switching to IgG1 or IgE to similar extents and were mostly CD138^–^ (Figure 3—figure supplement 1a). It is of note that the iGB-lo cells expressed Fas at a lower level than iGB-hi and iGB-mid cells, whereas they expressed CD38 and CD62L at higher levels than iGB-hi and iGB-mid cells, perhaps reflecting their relatively lower activation status (Figure 3—figure supplement 1b).

**Figure 3—figure supplement 1.**
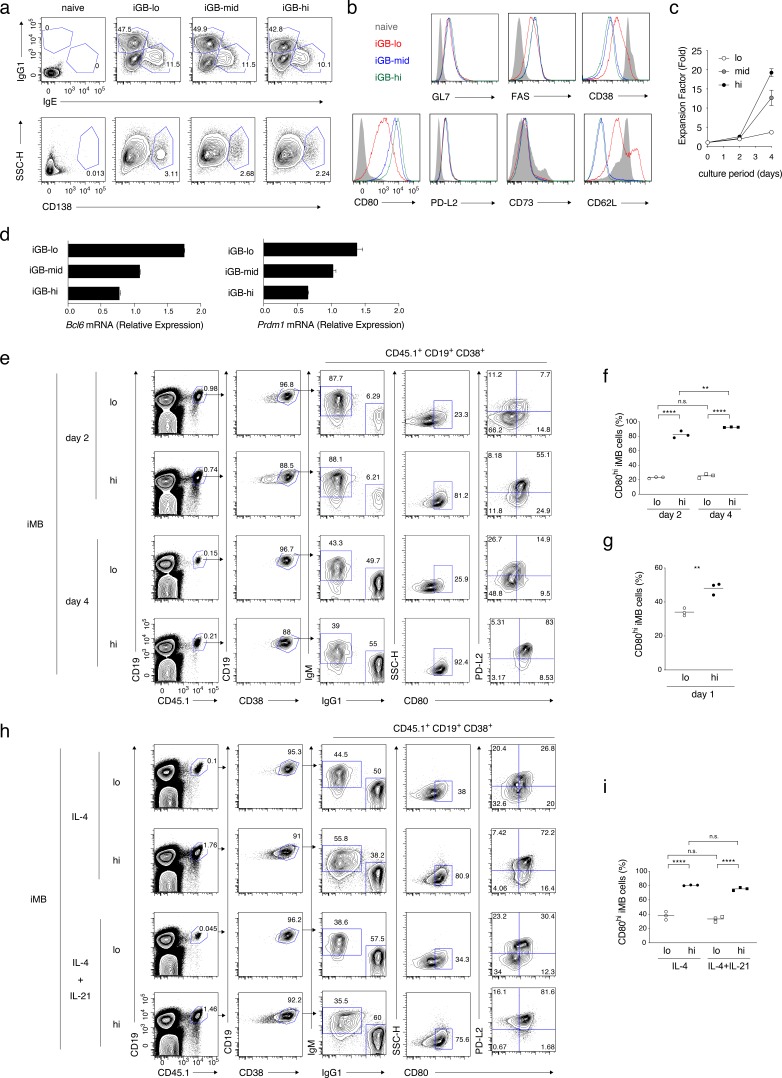
Cellular phenotype, proliferation capacity, gene expression, and differentiation fate of each type of iGB cells. (**a**) The frequencies of IgG1^+^ vs IgE^+^ cells, or of CD138^+^ cells, in naïve and the indicated iGB cells on day 4 of culture. (**b**) The expression of the indicated protein on naïve and the indicated iGB cells. (**c**) Cumulative fold increasein the numbers of B cells cultured on the feeder layer of each 40LB subline (hi, mid, lo). (**d**) qRT-PCR analysis of the expression of *Bcl6* and *Prdm1* mRNAs in the indicated iGB cells (mean + s.d. of triplicates). (**e, f**) iGB-lo and iGB-hi cells on day 2 or day 4 of the culture were transferred into irradiated B6 mice to generate iMB cells. Two weeks later, spleen cells of the recipient mice were analyzed by FCM. (**e**) A representative FCM data showing the gating strategy. (**f**) The frequency (%) of CD80^hi^ cells in iMB cells (CD19^+^ CD45.1^+^ CD38^+^) derived from the iGB-lo and iGB-hi cells on day 2 or day 4 (n = 3). (**g**) The frequency (%) of CD80^hi^ cells in iMB cells derived from iGB cells cultured for 1 day on 40LB-lo or 40LB-hi feeder cells (n = 3). (**h, i**) iMB cells were generated from iGB-lo and iGB-hi cells that were cultured with IL-4 and IL-21, or IL-4 alone, for 4 days. (**h**) Representative FCM data showing the gating strategy. (**i**) The frequency (%) of CD80^hi^ cells among the iMB cells derived from the iGB cells cultured in the indicated condition.

These iGB cells were then transferred into irradiated mice and, 2 weeks later, B cells in the spleen were analyzed by FCM (Figure 3c). The B cells derived from all of these iGB cells were mostly CD38^+^ (iMB) cells and contained similar percentages of IgG1^+^ cells. However, the iMB cells derived from iGB-hi cells (iMB-hi cells) contained almost exclusively CD80^hi^ cells, whereas iMB cells from iGB-lo cells (iMB-lo cells) were dominated by CD80^lo^ cells, irrespective of their BCR isotypes (IgG1^+^ or IgG1^–^). iMB cells derived from iGB-mid cells exhibited an intermediate phenotype (Figure 3d,e). In addition, iMB-hi cells expressed PD-L2, CD73 and FAS at higher levels than iMB-lo cells, whereas both expressed equally low levels of GL7 (Figure 3f). Thus, iMB-hi and iMB-lo cells phenotypically resembled CD80^hi^ and CD80^lo^ B_mem_ cells, respectively, that are generated in a physiological immune response (Figure 1a).

iGB-hi cells grow far more extensively than iGB-lo cells beyond two days of culture (Figure 3—figure supplement 1c). As cell cycling may cause epigenetic and transcriptional changes, it is possible that different cell cycles caused by different CD40 signaling strength affected the bifurcated B_mem_ cell development. To address this issue, iMB cells were generated by transferring iGB-lo and iGB-hi cells on day 2 of culturing, when these cells had expanded to similar levels. As a result, about 80% of iMB cells derived from the day 2 iGB-hi cells were CD80^hi^, whereas about 20% from iGB-lo cells were CD80^hi^. These results are reminiscent of those from iMB cells generated from the day 4 iGB cells, except that the iMB cells from day 2 iGB cells contained fewer IgG1^+^ and fewer PD-L2^+^ cells (Figure 3—figure supplement 1e,f). iGB-hi cells, rather than iGB-lo cells, tended to dominate the generation of CD80^hi^ iMB cells, even when we used day 1 iGB cells (Figure 3—figure supplement 1g). These data indicate that the bifurcated B_mem_ cell fate was not determined by different levels of cell proliferation. However, they also demonstrated that the frequency of the CD80^hi^ population in iMB-hi cells increased as the culture period of iGB-hi cells became longer (i.e. from 1 to 2 to 4 days), thus indicating that the duration of CD40 signaling may also affect the development of CD80^hi^ B_mem_ cells. Although IL-21 is a hallmark T_FH_ cytokine, which is known to support GC B cell proliferation, the addition of IL-21 to the iGB-hi and iGB-lo cell culture did not affect the frequencies of CD80^hi^ iMB cells derived from each type of iGB cells (Figure 3—figure supplement 1h,i), suggesting that the contribution of IL-21 to the differential B_mem_ cell fate decision in vivo is less likely.

**Figure 4.**
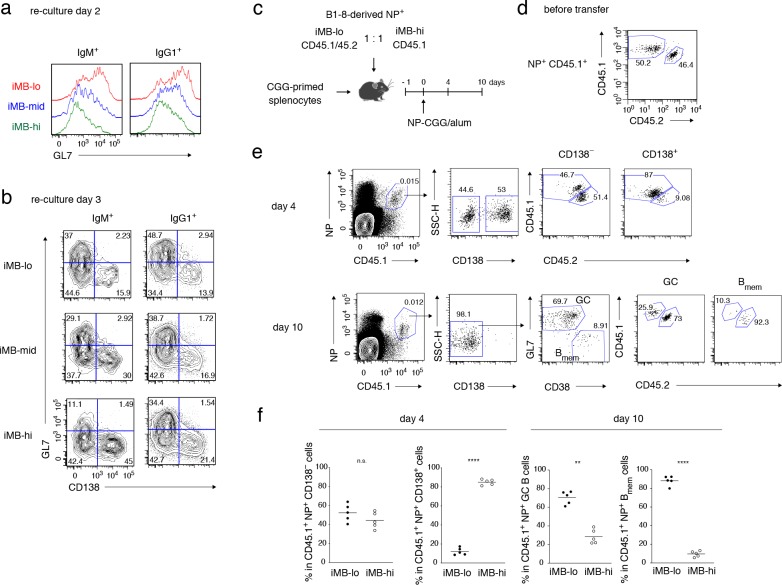
Primary CD40 signaling strength affects secondary B_mem_ cell differentiation, either to PCs or to GC B cells. (**a, b**) Splenic B cells from each recipient mouse (containing iMB cells), generated as in Figure 3 (**d**), were cultured on 40LB feeder layers with IL-21 for 2 (**a**) or 3 (**b**) days. The expression of GL7 (**a, b**) and CD138 (**b**) on gated IgM^+^ or IgG1^+^ CD45.1^+^ (iMB cell-derived) cells was analyzed by FCM. (**c–f**) iMB-lo and iMB-hi cells were generated from B1-8 ki Igκ^−/−^ CD45.1/CD45.2 or B1-8 ki Igκ^−/−^ CD45.1 iGB cells, respectively, as in Figure 3 (**d**). 2.5 × 10^4^ (for ‘day 4’) or 1 × 10^4^ (for ‘day 10’) of NP^+^ iMB-lo and iMB-hi cells were mixed and co-transferred into WT B6 recipient mice with 1 × 10^7^ CGG-primed splenocytes. The recipient mice were then immunized with NP-CGG in alum and analyzed 4 or 10 days after immunization. (**c**) A schematic of the experimental procedure. (**d**) A representative FCM profile of the mixture of iMB-lo (CD45.2^+^) and iMB-hi (CD45.2^−^) cells, gated on CD45.1^+^ NP^+^ cells, before the transfer. (**e**) Representative FCM data at day 4 and day 10 after immunization showing the gating strategy. (**f**) The frequencies of iMB-lo- and iMB-hi-derived cells among CD45.1^+^ NP^+^ CD138^−^ or CD138^+^ cells at day 4, and among CD45.1^+^ NP^+^ B_mem_ cells (CD138^−^ GL7^−^ CD38^+^) or GC B cells (CD138^−^ GL7^+^ CD38^−^) at day 10. The mean of the values in each group is indicated by a horizontal bar (**f**). n.s., not significant (p>0.05); **, p<0.01; ****, p<0.0001; as determined by paired Student’s *t* tests (**f**). All data are representative of two independent experiments. 10.7554/eLife.44245.013Figure 4—source data 1.Source data for Figure 4f.

In order to examine whether the iMB-hi and iMB-lo cells recapitulate the functional differences seen in CD80^hi^ and CD80^lo^ B_mem_ cells, we analyzed their differentiation in culture on the 40LB feeder cells with IL-21. Regardless of their BCR isotype, iMB-lo cells preferentially differentiated toward GC B cells, as clearly seen on day 2, whereas iMB-hi cells preferentially differentiated into plasmablasts or PCs, becoming evident on day 3. The behavior of the iMB-mid cells was intermediate (Figure 4a,b and Figure 4—figure supplement 1). Next, we investigated the in vivo fate of these iMB cells in response to immunization with a cognate antigen. We sorted allotypically marked NP-binding iMB-lo and iMB-hi cells, derived from iGB-lo and iGB-hi cells of B1-8ki Igκ^−/−^ mice, respectively, and co-transferred the iMB-lo and the iMB-hi cells at an equal ratio into B6 mice together with carrier (CGG)-primed T cells. The recipient mice were immunized with NP-CGG, and their spleen cells were analyzed 4 or 10 days later by FCM (Figure 4c,d). Four days after immunization, most donor-derived, NP-binding plasmablasts were derived from iMB-hi cells. By contrast, the vast majority of donor-derived, NP-binding GC B and B_mem_ cells at day 10 were found to originate from iMB-lo cells (Figure 4e,f). These in vitro and in vivo data together indicate that iMB-hi and iMB-lo cells functionally represent CD80^hi^ and CD80^lo^ B_mem_ cells, respectively. Taken together, these data indicate that the quantity of CD40 signaling in B cells determines their differentiation fate toward phenotypically and functionally distinct B_mem_ cell subsets.

**Figure 4—figure supplement 1.**
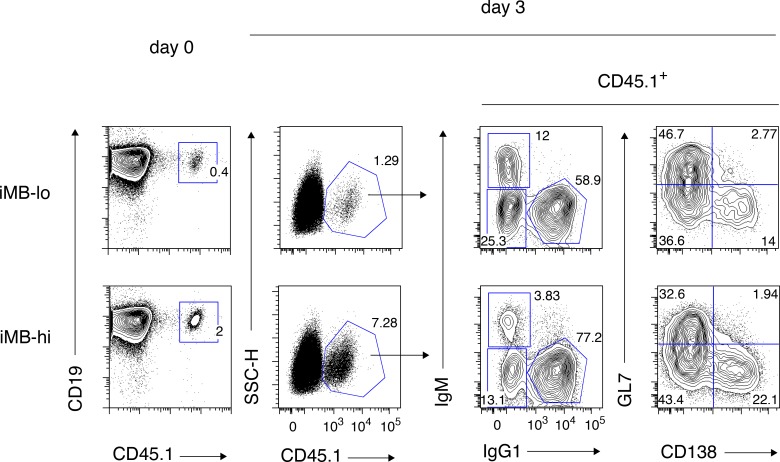
A gating strategy for IgM+ and IgG1+ cells used in Figure 4b. Spleen cells containing donor-derived (CD45.1^+^) iMB cells (left) were cultured as shown in Figure 4a,b. FCM profiles of the gated iMB cell-derived cells on day 3, with the gating strategy for the cell fractions used in Figure 4b, are shown (right). The analysis on day 2 in Figure 4a was gated in the same manner.

### Higher BCR affinity for antigen favors the development of CD80^hi^ B_mem_ cells

**Figure 5.**
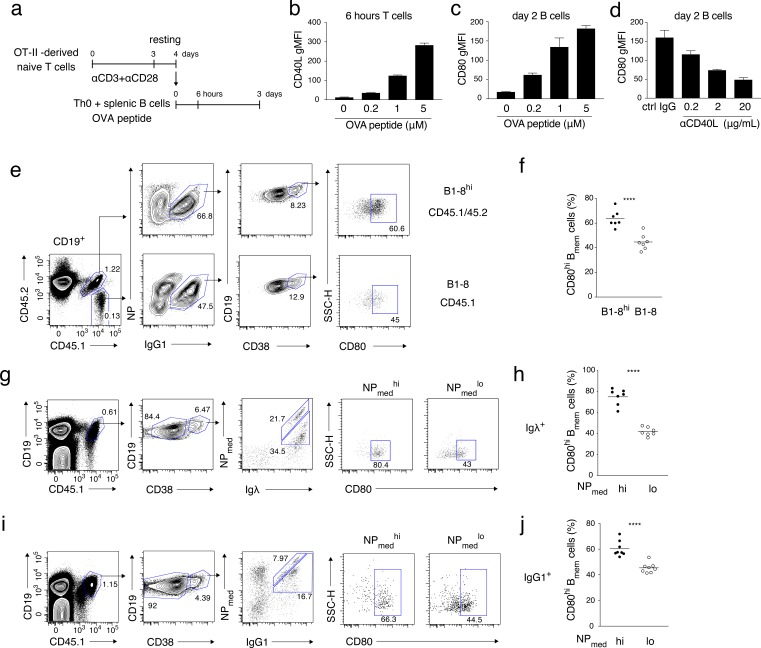
High-affinity B cells preferentially differentiate into CD80^hi^ B_mem_ cells, possibly through stronger induction of CD40L on cognate T cells. (**a–d**) OT-II-derived Th0 cells and splenic B cells were co-cultured with the indicated concentration of OVA peptide for 6 hr or 2 days and analyzed by FCM. (**a**) An outline of the procedure for the T-B co-culture (see Materials and methods). (**b**) Expression levels of CD40L on CD4^+^ T cells after 6 hr co-culture are presented as gMFI (mean + s.d. of triplicates). (**c**) Expression levels of CD80 on CD19^+^ B cells after 2 days co-culture are presented as gMFI (mean + s.d. of triplicates). (**d**) Expression levels of CD80 on B cells after 2 days co-culture with 5 μM OVA peptide in the presence of the indicated concentration of anti-CD40L blocking Ab. Data are shown as in (**c**). (**e, f**) 1 × 10^5^ NP^+^ splenic B cells from B1-8^hi^ ki (CD45.1/45.2) or B1-8 ki (CD45.1) mice were co-transferred into the recipient B6 mice, which were immunized with NP-CGG in alum on the next day and analyzed by FCM 7 days later. (**e**) Representative FCM data showing the gating strategy. (**f**) The frequency (%) of CD80^hi^ among IgG1^+^ B_mem_ cells (CD19^+^ CD38^+^) derived from either B1-8^hi^ ki or B1-8 ki cells is plotted (n = 7). (**g–j**) B6 mice transferred with B1-8 ki B cells and immunized as in (**e, f**) were analyzed by FCM at 10 days after immunization. (**g, i**) Representative FCM data showing the gating strategy. (**h, j**) The frequencies (%) of CD80^hi^ cells among NP_med_^hi^ or NP_med_^lo^ cells in Igλ^+^ (**h**) or IgG1^+^ (**j**) B_mem_ cells (CD19^+^ CD45.1^+^ CD38^+^), gating of each as shown in (**g**) and (**i**), respectively (n = 8). The mean of the values in each group is indicated by a horizontal bar (**f, h, j**). ****, p<0.0001; as determined by paired Student’s *t* tests (**f, h, j**). All data are representative of two independent experiments except (**f, h, j**), where data from two independent experiments are combined. 10.7554/eLife.44245.016Figure 5—source data 1.Source data for Figure 5b, c, d, f, h, j.

On the basis of the data described above, it is likely that the CD40 signaling quantity is primarily determined by the expression level of CD40L on cognate T cells. As CD40L on T cells was shown to be induced in an antigen-dose-dependent manner (Jaiswal and Croft, 1997), it seemed plausible that the quantity of antigen presented on B cells would determine the expression level of CD40L on cognate T cells, which in turn determines the differentiation fate toward each B_mem_ subset. To confirm that antigen presentation by B cells induces CD40L on cognate T cells in a dose-dependent manner, OT-II-mouse-derived activated T cells were co-cultured with B cells and various concentrations of OVA peptide (Figure 5a and Figure 5—figure supplement 1). CD40L expression on the T cells was rapidly induced and its levels positively correlated with antigen dose (Figure 5b), as was also the case for CD80 expression on B cells on day 2 (Figure 5c). This CD80 induction was suppressed by blocking with anti-CD40L mAb, confirming that the CD40L–CD40 interaction leads to CD80 induction on B cells (Figure 5d).

**Figure 5—figure supplement 1.**
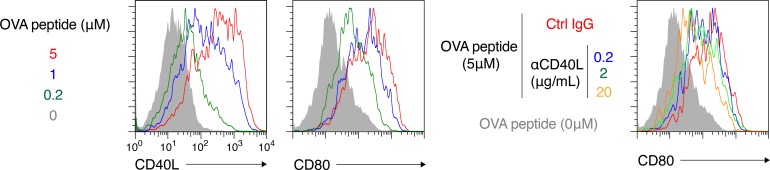
Representative FCM profile data from the analyses shown in Figure 5b–d.

During an immune response, B cells expressing a high-affinity BCR would take up more of the cognate antigen, and thus would present a larger amount of antigenic peptide–MHC complex to cognate T cells (Schwickert et al., 2011). This would lead to greater CD40L induction than would occur on T cells interacting with B cells with a lower affinity BCR. To investigate the correlation between BCR affinity and B cell differentiation fate, we used B1-8^hi^ ki mice whose λ^+^ B cells express BCR with ten-fold higher NP affinity than BCR expressed on λ^+^ B1-8 ki B cells (Allen et al., 1988). B cells from B1-8^hi^ ki and B1-8 ki mice, expressing discriminating allotypic markers, were co-transferred into B6 mice, and immunized with NP-CGG in alum. FCM analysis on day 7 after the immunization revealed that IgG1^+^ B_mem_ cells developed from B1-8^hi^ ki B cells contained a higher frequency of CD80^hi^ cells than those from B1-8 ki B cells (Figure 5e,f).

In the next experiment, we stained B_mem_ cells with NP_med_-APC, allophycocyanin (APC) conjugated with NP at a relatively lower valency, which only binds to high affinity anti-NP BCRs (Nishimura et al., 2011). Mice were transferred with B1-8 ki B cells, immunized with NP-CGG in alum, and analyzed by FCM 10 days later. Among donor-derived Igλ^+^ or IgG1^+^ B_mem_ cells, those stained more brightly with NP_med_ (NP_med_^hi^) contained a higher frequency of CD80^hi^ B_mem_ cells than those stained less brightly (NP_med_^lo^) (Figure 5g–j). These data indicated that B cells with higher antigen affinity preferentially differentiate into CD80^hi^ B_mem_ cells rather than CD80^lo^ B_mem_ cells, probably through more extensive antigen presentation to cognate T cells, which results in greater induction of CD40L. Alternatively, B cells with lower affinity may be excluded from the GC and therefore fail to access to CD40L on T_FH_ cells. In any case, BCR affinity appears to be a primary determinant for the differential B_mem_ subset development that is dependent on CD40 signaling quantity.

### Possible mechanisms through which CD40 signaling facilitates GC B-cell differentiation into CD80^hi^ B_mem_ cells

**Figure 6.**
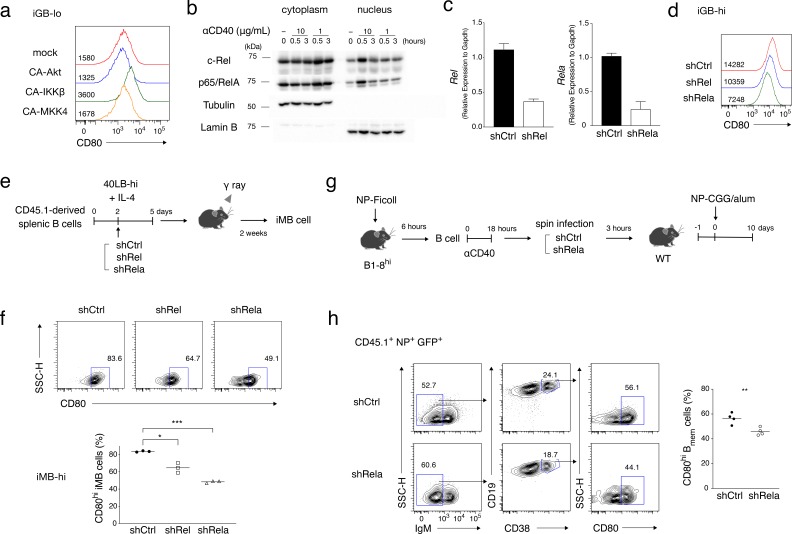
NF-κB signaling is involved in CD80^hi^ B_mem_ cell development. (**a**) Constitutively active (CA) variants of Akt, IKKβ or MKK4 were retrovirally transduced into B cells cultured on 40LB-lo feeder cells (iGB-lo cells) on day 2 of the culture. The expression of CD80 on the gated IgG1^+^ CD138^−^ infection-marker-positive iGB-lo cells was then analyzed by FCM on day 5. The number on each histogram indicates gMFI. (**b**) Cytoplasmic and nuclear lysates from B cells stimulated with CD40 mAb (1 or 10 mg/ml) for the indicated time periods were analyzed by immunoblotting using Abs against c-Rel and p65/RelA. Tubulin and Lamin B were used as loading controls for cytoplasmic or nuclear proteins, respectively. (**c**) B cells cultured on 40LB-hi feeder cells (iGB-hi cells) were transduced with shCtrl, shRel, or shRela retroviral vectors, each carrying a GFP gene as an infection marker. Three days after the transduction, the expression of *Rel* and *Rela* mRNA in the sorted GFP^+^ cells was analyzed by qRT-PCR (mean + S.D. of triplicates). (**d**) Expression of CD80 on the GFP^+^ IgG1^+^ CD138^−^ iGB-hi cells analyzed by FCM at 3 days after gene transduction, as in (**c**). (**e, f**) The iGB-hi cells transduced with the knock-down constructs as shown in (**c,d**) were transferred into γ-irradiated mice, spleens of which were analyzed by FCM 2 weeks later. (**e**) Outline of the experimental procedure. (**f**) Representative FCM data showing the the expression of CD80 (above) and the frequency (%) of CD80^hi^ cells (bottom; n = 3) in the gene-transduced iMB cells (CD19^+^ CD45.1^+^ CD38^+^ GFP^+^) formed in the recipients’ spleens. (**g, h**) In vivo activated B cells derived from B1-8^hi^ ki mice were transduced with shCtrl or shRela vectors, as described in the Materials and methods, and the resultant B cells (1 × 10^6^) were transferred into WT B6 mice. The recipient mice were immunized with NP-CGG in alum on the next day. Splenocytes from these mice were analyzed by FCM at 10 days after immunization. (**g**) Outline of the experimental procedure. (**h**) Representative FCM data showing the gating strategy (left). The frequencies (%) of CD80^hi^ cells among donor-derived, vector-transduced, and class-switched B_mem_ cells (CD45.1^+^ NP^+^ GFP^+^ IgM^−^ CD19^+^ CD38^+^) at 10 days after immunization (right; n = 4). The mean of the values in each group is indicated by a horizontal bar (**f, h**). *, p<0.05; **, p<0.01; ***, p<0.001; as determined by unpaired Student’s *t* tests. All data are representative of two independent experiments. 10.7554/eLife.44245.019Figure 6—source data 1.Source data for Figure 6c, f and h.

We next investigated the CD40 signaling mechanisms that are responsible for the development of the CD80^hi^ B_mem_ cell subset. NF-κB is a typical transcription factor that are induced by CD40 stimulation (Berberich et al., 1994), and the p50/p65 heterodimer was reported to bind to the *Cd80* gene locus and to induce CD80 expression in a B cell line after stimulation (George et al., 2006). In accord with these data, among the constitutively active (CA) forms of protein kinases that are known to be activated by CD40 stimulation, CA-IKKβ, an activator of the canonical NF-κB pathway (Mercurio et al., 1997), but not CA-Akt or CA-MKK4, upregulated CD80 expression on iGB-lo cells (Figure 6a). Stimulation of splenic B cells with a higher concentration (10 μg/ml), but not a lower concentration (1 μg/ml), of anti-CD40 Ab induced the nuclear translocation of the NF-κB subunits c-Rel and RelA (Figure 6b). Furthermore, knockdown of *Rel* and *Rela* gene expression in iGB-hi cells resulted in downregulation of surface CD80 expression (Figure 6c,d and Figure 6—figure supplement 1a). These data indicated that CD80 expression on B cells induced by strong CD40 signaling is mediated by NF-κB, c-Rel and RelA. We next examined whether a similar signaling pathway is involved in the generation of B_mem_ cells. By using the iGB cell system, we showed that c-Rel- and Rela-knockdown iGB-hi cells generated fewer CD80^hi^ iMB cells than mock-treated iGB-hi cells in vivo (Figure 6c–f and Figure 6—figure supplement 1b). Furthermore, we transferred NP-specific B cells transduced with the *Rela*-knockdown vector or a mock vector into mice, which were then immunized with NP-CGG. The knockdown of *Rela* selectively suppressed the development of CD80^hi^ B_mem_ cells among the B cells that had responded to an immunized antigen (Figure 6g,h and Figure 6—figure supplement 1c). These data indicated that canonical NF-κB signaling plays a role in CD80^hi^ B_mem_ cell development that is facilitated by stronger CD40 stimulation.

**Figure 6—figure supplement 1.**
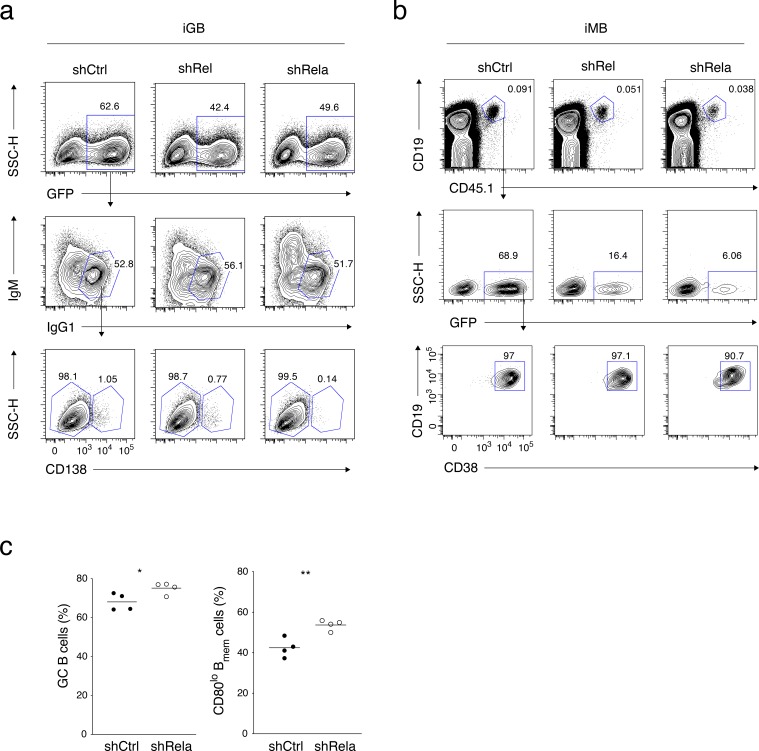
Gating strategies for the analyses in Figure 6d and f, and comlementary data for Figure 6h. (**a**) A representative FCM data showing the gating strategy for the gene-transduced IgG1^+^ CD138^−^ iGB cells analyzed in Figure 6d. (**b**) Representative FCM data showing the gating strategy for the gene-transduced iMB cells analyzed in Figure 6f. (**c**) The frequencies of GC B cells (CD19^+^ CD38^−^) in the donor-derived, antigen-specific, class-switched, gene-transduced B cells (CD45.1^+^ NP^+^ IgM^−^ GFP^+^) (left), and the frequencies of CD80^lo^ cells in the gene-transduced B_mem_ cells (CD45.1^+^ NP^+^ GFP^+^ IgM^−^ CD19^+^ CD38^+^), for the mice shown in Figure 6h (n = 4).

**Figure 7.**
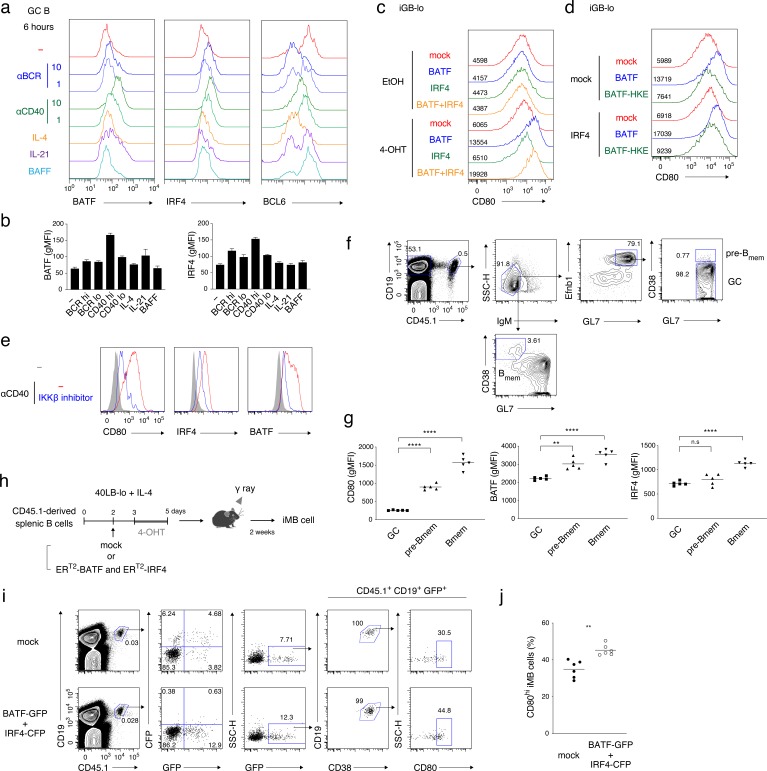
CD40-induced BATF may be involved in CD80^hi^ B_mem_ cell development. (**a, b**) GC B cells (CD19^+^ CD138^−^ CD38^−^ GL7^+^) were sorted from splenocytes of mice at 7 days after immunization with NP-CGG in alum and cultured without (−) or with the addition of the following reagents for 6 hr: anti-IgM plus anti-IgG Abs (αBCR, 10 μg/ml or 1 μg/ml), anti-CD40 Ab (αCD40, 10 μg/ml or 1 μg/ml), IL-4 (10 ng/ml), IL-21 (10 ng/ml) or BAFF (10 ng/ml). Expression of the indicated proteins in these B cells (CD19^+^ CD138^−^) was analyzed by intracellular staining followed by FCM. (**a**) Representative FCM data. (**b**) gMFIs of the histograms shown in (**a**). Data are mean + s.d. of triplicates. (**c**) Splenic B cells cultured on 40LB-lo feeder cells (iGB-lo) were transduced with a mock vector or with the indicated vectors expressing each factor fused with ER^T2^ (generated as described in the Materials and methods) on day 2 of the culture, and then treated with vehicle (EtOH) alone or with 4-OHT from day 3 to day 5. The expression of CD80 on these cells was analyzed on day 5, and shown as in (**a**). (**d**) iGB-lo cells were transduced with the indicated combination of the ER^T2^–fusion vectors and treated with 4-OHT as in (**c**). The CD80 expression on these cells is shown as in (**c**). (**e**) Splenic B cells were cultured with anti-CD40 Ab (20 μg/ml) for 2 days without (−) or with an IKKβ inhibitor (BAY11-7082). Expression of the indicated proteins in these cells was analyzed by FCM. The shadowed histograms represent the cells cultured with medium alone. (**f, g**) Splenic B cells from B1-8 ki CD45.1 mice were transferred into WT B6 mice, which were immunized with NP-CGG in alum on the next day. At 10 days after immunization, splenocytes from the recipients were analyzed by FCM. (**f**) A representative data showing the gating strategy. (**g**) gMFI of the indicated proteins in the donor-derived GC B cells (CD19^+^ CD45.1^+^ IgM^−^ GL7^+^ Ephrin B1^+^ CD38^−^), pre-B_mem_ cells (CD19^+^ CD45.1^+^ IgM^−^ GL7^+^ Ephrin B1^+^ CD38^+^), and B_mem_ cells (CD19^+^ CD45.1^+^ IgM^−^ GL7^−^ CD38^+^) (n = 5). (**h–j**) iGB-lo cells were transduced with the retroviral vectors expressing ER^T2^-BATF-ires-GFP and ER^T2^-IRF4-ires-CFP (BATF-GFP +IRF4 CFP), or with empty vectors expressing GFP and CFP (mock) on day 2 of culture treated with 4-OHT from day 3 to day 5, and then transferred into γ-irradiated mice. Two weeks after the transfer, spleen cells of the recipient mice were analyzed by FCM. (**h**) Outline of the experimental procedure. (**i**) A representative data showing the gating strategy. (**j**) The frequency (%) of CD80^hi^ cells in the ER^T2^-BATF gene-transduced iMB cells formed in the recipients’ spleens (CD19^+^ CD45.1^+^ CD38^+^ GFP^+^) (n = 6). The mean of the values in each group is indicated by a horizontal bar (**g, j**). *, p<0.05; **, p<0.01; as determined paired Student’s *t* tests. All data are representative of two independent experiments except (**j**), in which data from two independent experiments are combined. 10.7554/eLife.44245.022Figure 7—source data 1.Source data for Figure 7b, g and j.

It has been observed that CD40 stimulation induces IRF4 in B cells and that bone marrow-derived dendritic cells from IRF4-deficient mice express a reduced level of CD80 upon LPS stimulation (Saito et al., 2007; Suzuki et al., 2004). It was also reported that transient or intermediate expression of IRF4 induced GC-related genes through the formation of heterodimers with BATF or PU.1, whereas its sustained or high expression induced PC-related genes through an IRF4 homodimer (Ochiai et al., 2013). Thus, we examined whether these transcription factors are involved in CD40 signaling in GC B cells. When ex-vivo GC B cells were cultured with anti-CD40 or anti-BCR Abs, of either high or low doses, or with various cytokines, IRF4 expression was found to be upregulated by a high dose of anti-CD40 or anti-BCR Abs, whereas BATF expression was selectively upregulated by a high dose of anti-CD40 Ab (Figure 7a,b and Figure 7—figure supplement 1a). Then, we tested whether BATF and IRF4 are involved in the induction of CD80 by using tamoxifen-inducible ER^T2^-BATF or ER^T2^-IRF4 constructs. Induced activation of BATF selectively upregulated CD80 expression in iGB-lo cells, although IRF4 alone did not, and co-activation of BATF and IRF4 slightly enhanced CD80 expression (Figure 7c and Figure 7—figure supplement 1b,c). In addition, a BATF mutant (BATF- HKE), which is defective in IRF4 binding (Tussiwand et al., 2012), failed to upregulate CD80 expression regardless of exogenous IRF4 (Figure 7d), suggesting that the exogenous BATF formed a heterodimer with endogenous IRF4 for CD80 upregulation. These data together indicate that the BATF–IRF4 heterodimer that is induced by strong CD40 signaling enhances CD80 expression in activated B cells. As an IKKβ inhibitor suppressed CD40-induced expression of CD80 as well as of BATF and IRF4, the canonical NF-κB pathway appears to upregulate the expression of BATF and IRF4 (Figure 7e).

**Figure 7—figure supplement 1.**
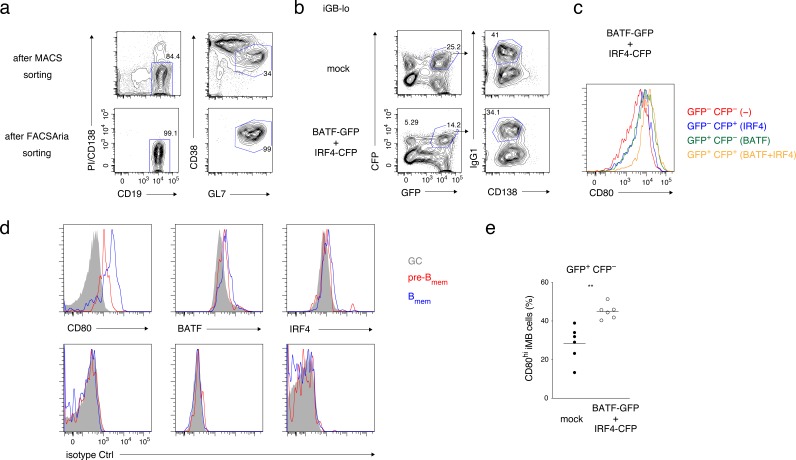
Supplementary data for Figure 7. (**a**) GC B cells magnetically enriched from splenocytes of the mice immunized 1 week previously (top) and further purified by fluorescence-activated cell sorting (FACS) sorting (bottom) were used for the analyses in Figure 7a. (**b**) Representative FCM data of iGB-lo cells on day 5 of the culture shown in Figure 7h. (**c**) The expression of CD80 on the indicated populations of IgG1^+^ iGB-lo cells shown in (**b**). (**d**) A representative FCM data for the analyses in Figure 7g. Cells were stained extra- (CD80) or intra-cellularly (BATF, IRF4) with antibodies against the indicated proteins (top) or with isotype-matched control antibodies (bottom). (**e**) The frequency of CD80^hi^ B_mem_ cells in GFP^+^ CFP^−^ fractions of iMB cells, as defined in the second panels from the left in Figure 7i, and in the experiment in Figure 7h–j. Legend for Supplementary file 1.

Considering that the NF-κB pathway upregulates CD80 expression on iGB cells and facilitates CD80^hi^ B_mem_ cell development in vivo (Figure 6), it is possible that the BATF–IRF4 heterodimer plays a role in the strong CD40 signal that drives GC B cell differentiation into CD80^hi^ B_mem_ cells. This idea was supported by our finding that GL7^+^ Efnb1^+^ CD38^+^ GC-derived memory precursors (pre-B_mem_) cells (Laidlaw et al., 2017) expressed BATF and CD80 at higher levels than GL7^+^ Efnb1^+^ CD38^−^ GC B cells at 10 days after immunization (Figure 7f,g and Figure 7—figure supplement 1d).

Finally, to investigate whether the BATF–IRF4 heterodimer is involved in the development of CD80^hi^ B_mem_ cells, iMB cells were generated from iGB-lo cells transduced with both ER^T2^-BATF and ER^T2^-IRF4, which were then activated in the culture with 4-OHT (Figure 7h). In the iMB cells, ER^T2^-IRF4-expressing (CFP^+^) cells could not be detected, possibly because cells that expressed an excess amount of IRF4 had differentiated into PCs. On the other hand, ER^T2^-BATF-expressing (GFP^+^) iMB cells were present and contained a significantly higher proportion of CD80^hi^ cells than mock-transduced iMB cells (Figure 7i,j and Figure 7—figure supplement 1e). Taken together, our data indicate that strong CD40 signaling is converted into the activation of NF-κB and the following upregulation of BATF, resulting in the generation of CD80^hi^ B_mem_ cells.

## Discussion

The regulation of the bidirectional response of B_mem_ cells, either to PCs or to GC B cells upon secondary antigen challenge, has recently been explained by defining the functionally different B_mem_ cell subsets. According to the report by Shlomchik and colleagues, CD80^+^ PD-L2^+^ B_mem_ cells preferentially differentiate into PCs, whereas CD80^−^ PD-L2^+^ and CD80^−^ PD-L2^−^ B_mem_ cells differentiate to GC B cells (Zuccarino-Catania et al., 2014). As the class-switched B_mem_ cell population that we mainly focused on is mostly composed of CD80^+^ PD-L2^+^ and CD80^−^ PD-L2^+^ cells, and also because CD80^−^ PD-L2^+^ and CD80^−^ PD-L2^−^ B_mem_ cells are functionally similar to each other (Zuccarino-Catania et al., 2014), we reasoned that the proposed subsets can simply be distinguished by CD80 expression as CD80^hi^ and CD80^lo^ B_mem_ cells, a distinction that we applied in this study to make a multi-color FCM analyses easier. The CD80^hi^ B_mem_ cells were mostly PD-L2^+^, CD73^+^, CD62L^lo^, whereas CD80^lo^ B_mem_ cells included PD-L2^+^ and PD-L2^–^ cells, CD73^+^ and CD73^–^ cells, and mostly CD62L^hi^, which was largely consistent with previous reports (Dogan et al., 2009; He et al., 2017; Pape et al., 2011; Zuccarino-Catania et al., 2014).

Our data showing preferential differentiation of the CD80^hi^ and CD80^lo^ B_mem_ cells into plasmablasts/PCs and GC B cells in vitro, respectively, was also consistent with the previous in vivo data showing the preferential differentiation of each B_mem_ cell subset during the recall response. Reports showing that B_mem_ cells corresponding to the CD80^hi^ B_mem_ cells have BCRs with higher antigen affinity than those corresponding to the CD80^lo^ B_mem_ cells, and that high affinity B cells are more prone to become PCs, seemed to suggest that BCR-signal strength determines the fates of CD80^hi^ and CD80^lo^ B_mem_ cells upon the secondary challenge (Phan et al., 2006; Zuccarino-Catania et al., 2014). However, our in vitro culture system without specific antigens clearly showed that the distinct fates of these B_mem_ subsets upon re-stimulation are not determined by the BCR affinity or isotype, although they might be affected by slightly different levels of CD40 expression on CD80^hi^ and CD80^lo^ B_mem_ cells (Figure 2—figure supplement 1j). Thus, cell status represented by, for example, transcriptomic or epigenetic profiles may largely define the function of each B_mem_ subset (He et al., 2017; Kometani et al., 2013; Zuccarino-Catania et al., 2014).

Previously it was reported that GC depletion by anti-CD40L mAb treatment reduced the frequency of CD80^hi^ B_mem_ cells, and that the transcriptomic signature of CD80^hi^ B_mem_ cells more closely correlated with CD40-stimulated B cells than did that of CD80^lo^ B_mem_ cells (He et al., 2017; Weisel et al., 2016). We demonstrated that the development of CD80^hi^ B_mem_ cells was largely dependent on the presence of T_FH_ cells, which express CD40L at a markedly higher level than naïve or effector T cells, and that partial blocking by anti-CD40L Ab or knock-down of CD40L on CD4 T cells during the primary response dominantly affected the generation of CD80^hi^ B_mem_ cells rather than CD80^lo^ B_mem_ cells. Conversely, in vivo stimulation with anti-CD40 Ab during the primary response increased the number of CD80^hi^ B_mem_ cells but not the numbers of CD80^lo^ B_mem_ cells nor GC B cells. As CD40L in the recipient mice was essential for the generation of both types of B_mem_ cells from transferred B cells, we hypothesized that the generation of either CD80^hi^ or CD80^lo^ B_mem_ cells is determined by a difference in the quantity of CD40 signaling.

This hypothesis was strongly supported by our simplified experimental system, which enables in vivo generation of memory-like B (iMB) cells without immunization from naive B cells cultured on feeder cells (40LB) and transferred into mice. Using 40LB cells expressing different levels of CD40L, we clearly showed that stronger in vitro stimulation via CD40 promoted the generation of CD80^hi^ iMB cells, whereas weaker stimulation facilitated the generation of CD80^lo^ iMB cells from B cells with essentially the same BCR repertoire of specificity and isotype. The CD80^hi^ and CD80^lo^ iMB cells phenocopied the CD80^hi^ and CD80^lo^ B_mem_ cells, respectively, in that CD80^hi^ iMB cells preferentially differentiate into plasmablasts/PCs and CD80^lo^ iMB cells into GC B cells (and B_mem_ cells in vivo) in ex-vivo culture and after antigen challenge in vivo. The commitment to differentiate into either CD80^hi^ or CD80^lo^ iMB cells, as determined by the different quantities of CD40 signaling in B cells, was made within two days of culture when the proliferation did not differ among the conditions, and partially made in just one day when CD80 expression was hardly detectable on B cells. Therefore, it seems that the quantity of CD40 signaling in B cells directs cellular programming, which determines the differentiation into distinct B_mem_ cell subsets.

**Figure 8.**
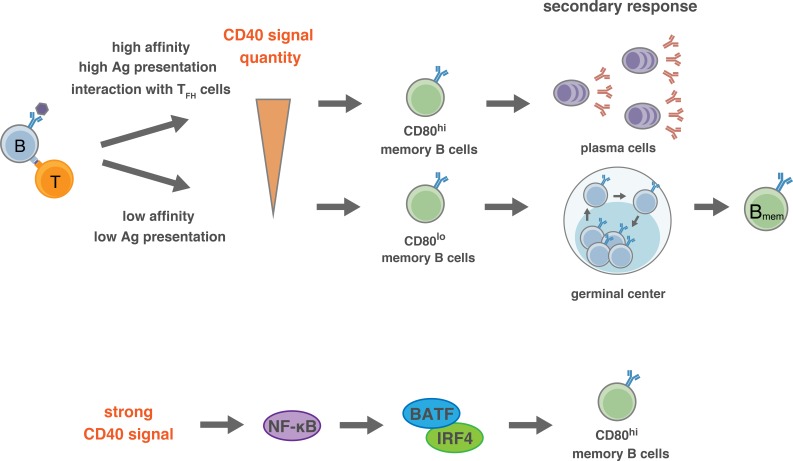
Proposed model for the generation of CD80^hi^ and CD80^lo^ B_mem_ cells. (Top) In the pre-GC phase of the primary response, the BCR affinity to antigen or the amount of available antigen determine the quantity of antigen presentation to T cells, and the extent of the induction of CD40L on T cells. Thus, the strength of CD40 signaling in B cells is determined by the interacting T cells, which then directs the differentiation fate to distinct B_mem_ subsets: relatively stronger CD40 signal commits B cells towards CD80^hi^ B_mem_ cells, whereas weaker CD40 signal commits B cells towards CD80^lo^ B_mem_ cells. After GC formation, T_FH_ cells, being able to express a high level of CD40L after TCR stimulation, strongly stimulate CD40 on relatively high-affinity B cells and facilitate their differentiation to CD80^hi^ B_mem_ cells. (Bottom) Activation of NF-κB, and the downstream BATF–IRF4 heterodimer, may transmit the strong CD40 signaling into a mechanism that facilitates the differentiation towards CD80^hi^ B_mem_ cells.

Although the B cells cultured on 40LB feeder cells (iGB cells) mimic some aspect of GC B cells, naturally they differ from genuine GC B cells in that iGB cells are uniformly proliferating, are CD80^+^ (to distinct levels depending on the strength of CD40 stimulation), and do not mutate Ig genes. We consider that the iGB cells that were primarily cultured with IL-4 may represent a certain state of T-cell-activated B cells that are destined to become B_mem_ cells, such as naïve B cells that are activated in the initial phase of primary response or GC B cells that have just undergone selection as what we call pre-B_mem_ cells. Therefore, our data describing the in vivo differentiation of iGB cells into B_mem_-like iMB cells probably explain the mechanism for the induction of bidirectional B_mem_ cell differentiation upon T-B interactions, during both the early phase and the GC phase of the primary immune response. Thus, in either phase, B cells receiving relatively stronger CD40 signaling are committed to CD80^hi^ B_mem_ cells, whereas those receiving weaker CD40 signaling are committed to CD80^lo^ B_mem_ cells. Our additional data suggest that, in the early phase, B cells expressing BCR with higher affinity to antigen present more antigenic peptide to cognate T cells, thus inducing CD40L on these T cells more strongly so that they acquire stronger CD40 signaling than lower affinity B cells. Similarly, in the GC phase, higher-affinity GC B cells dominantly present antigenic peptide, and will acquire more frequent and more durable interactions with T_FH_ cells that express a high level of CD40L (Figure 8).

Thus, stronger CD40 signaling in the GC may direct the development of CD80^hi^ B_mem_ cells, as supported by our data. Despite this supposition, it was reported that strong T cell help and CD40 signaling in vivo induce the differentiation of GC B cells into PCs (Ise et al., 2018; Schwickert et al., 2011), raising a question as to the mechanism for the differentiation of GC B cells into either CD80^hi^ B_mem_ cells or PCs. The fact that CD40 stimulation suppresses PC generation in vitro (Hawkins et al., 2013; Randall et al., 1998; Satpathy et al., 2010) suggests that the strong CD40 signaling alone does not directly promote PC differentiation in the GC. It is possible that additive BCR signaling affects the differentiation of GC B cells into PCs (Kräutler et al., 2017), although it has been reported that BCR signaling is inactive in most GC B cells (Khalil et al., 2012). Supposing that T_FH_ cells are heterogeneous in terms of cytokine production (Weinstein et al., 2016), a cytokine produced from a particular T_FH_ cell subset that interacts with the GC B cells may play a key role. Thus, a combination and integration of signaling pathways, one from the strong CD40 stimulation, the other from a particular cytokine, and maybe more, may ultimately determine the fate of GC B cells. IL-21 is known to induce B cell differentiation into PCs, while IL-21R-defficiency attenuated PC development and accelerated B_mem_ cell development (Zotos et al., 2010). In addition, we previously reported that iGB cells that were secondarily cultured with IL-21 preferentially develop into bone marrow PCs but not B_mem_ cells in vivo after adoptive transfer (Nojima et al., 2011). Thus, when GC B cells interact with IL-21-producing T_FH_ cells, and receive a strong CD40 signal, they will differentiate into PCs. A T_FH_ cell subset that induces the differentiation of GC B cells into CD80^hi^ B_mem_ cells has yet to be defined. IL-4-producing T_FH_ cells may be this subset, because iGB cells that are cultured with IL-4 on the CD40L^high^ feeder preferentially differentiated into CD80^hi^ iMB cells in vivo. Considering a report showing that B_mem_ cells develop from B cells in the earlier GC, whereas long-lived PCs are generated during the later GC (Weisel et al., 2016), it is possible that distinct T_FH_ subsets may work dominantly in B cell selection along the time course of the GC reaction.

Our data demonstrating that CD80^lo^ B_mem_ cell generation was little affected by the absence of GC resulting from T_FH_ cell-deficiency is consistent with a report indicating that the majority of CD80^lo^ PD-L2^–^ B_mem_ cell cells were generated prior to GC formation (Weisel et al., 2016). Given that low-affinity B cells do enter into the GC reaction and could maintain their low affinity even after mutation, why are only few CD80^lo^ B_mem_ cells generated during the GC phase? It has been shown that half of GC B cells undergo apoptosis every 6 hr (Mayer et al., 2017), and that this response can be avoided by CD40 signaling (Luo et al., 2018; Mayer et al., 2017). These data suggest that weaker CD40 signaling in the GC phase may not be enough to prevent the apoptosis of B cells and therefore could fail to induce CD80^lo^ B_mem_ cell development. Alternatively, lower-affinity B cells may be excluded from the GC in a competitive situation (Schwickert et al., 2011), and therefore fail to receive a strong CD40 stimulation from T_FH_ cells.

As mentioned earlier, CD80^hi^ and CD80^lo^ B_mem_ cells phenotypically and functionally resemble effector memory T (T_EM_) and central memory T (T_CM_) cells, respectively, in that T_EM_ cells are CD62L^–^ and produce abundant effector cytokines upon an antigen re-challenge, whereas T_CM_ cells are CD62L^+^ and have a greater potential for proliferation (Mueller et al., 2013). Our finding that CD40 signal strength directs the generation of CD80^hi^ or CD80^lo^ B_mem_ cells also resembles mechanistic aspects of the current model for the generation of T_EM_ and T_CM_ cells: stronger TCR signaling favors T_EM_ cells, whereas weaker TCR signaling favors the generation of T_CM_ cells (Daniels and Teixeiro, 2015). It has been reported that the commitment to CD4^+^ T_EM_ or T_CM_ cells is determined by the expression of T-bet and BCL6 transcription factors, respectively (Pepper et al., 2011), and that low-affinity TCR signaled greater induction of BCL6 expression but less expression of T-bet compared to high-affinity TCR (Knudson et al., 2013). Therefore, it is likely that TCR affinity/signal strength determines the direction of differentiation to distinct T_mem_ subsets, through the induction of distinctive transcription factors (Daniels and Teixeiro, 2015).

Previous studies and our observations imply transcriptomic predisposition of CD80^hi^ and CD80^lo^ B_mem_ cells that may account for their preferential differentiation upon re-stimulation, and suggest that the transcriptomic statuses may be established during the primary response as proposed for the T cell memory. It has been demonstrated that the CD40-NF-κB-IRF4 pathway represses the transcription of *Bcl6* (Saito et al., 2007), and that *Bcl6* mRNA is more abundantly expressed in CD80^−^ PD-L2^−^ cells than in CD80^+^ PD-L2^+^ cells (Zuccarino-Catania et al., 2014). Combined with our data suggesting that NF-κB and the downstream IRF4–BATF heterodimer play a role in generation of CD80^hi^ B_mem_ cells, the development to CD80^hi^ and CD80^lo^ B_mem_ cells is determined by the balance of the expression levels of transcription factors such as IRF4, BATF and BCL6, which are regulated by CD40 signaling quantity.

Canonical NF-κB mainly consists of heterodimer p50/c-Rel or p50/RelA (Jost and Ruland, 2017). It has recently been proposed that c-Rel and RelA have different roles in late B cell development: c-Rel promotes proliferation, whereas RelA upregulates Blimp1, a master regulator of PCs (Heise et al., 2014; Roy et al., 2019). Our data indicate that RelA is contributes more to the generation of CD80^hi^ iMB cells than does c-Rel, which may be involved in determining the nature of CD80^hi^ B_mem_ cells that are predisposed to PC development. Although RelA and c-Rel may function redundantly to some extent to generate CD80^hi^ B_mem_ cells, our data showing reduced generation of CD80^hi^ iMB cells by c-Rel knockdown alone also suggests a unique role for c-Rel.

What would be the survival advantage for individuals of generating bifurcated B_mem_ cell subsets upon infection? CD80^hi^ B_mem_ cells express high-affinity BCR (Zuccarino-Catania et al., 2014) and therefore produce high-affinity Abs. On the other hand, CD80^lo^ B_mem_ cells mainly express low-affinity BCRs, but have a greater proliferative potential and preferentially generate secondary GC upon reencounter with antigen, when they can further diversify their BCR repertoire. Therefore, CD80^lo^ B_mem_ cells, unlike high-affinity CD80^hi^ B_mem_ cells, can cope with a broader array of epitopes that may be generated by pathogens through mutations. Taken together, robust and rapid high-affinity Ab production by CD80^hi^ B_mem_ cells eliminates the majority of reinfecting pathogens, whereas pathogens that escape destruction because of epitope changes resulting from mutations are eliminated by Abs derived from CD80^lo^ B_mem_ cells that evolved in GCs. Thus, if we could dissect the CD40 signaling pathways that direct differentiation into CD80^hi^ B_mem_ cells and those promote cell proliferation or survival, selective suppression of the former pathways during vaccination might convert the generation of CD80^hi^ B_mem_ cells into CD80^lo^ B_mem_ cells that would eventually produce broadly reactive Abs. Alternatively, additive stimulation of CD40 with proper timing would facilitate the generation of CD80^hi^ B_mem_ cells that rapidly produce highly specific Abs upon actual infection.

## Materials and methods

**Key resources table:** 

Reagent type (species) or resource	Designation	Source or reference	Identifiers	Additional information
Genetic reagent (*M. musculus*)	B1-8ki	Lam et al., 1997		Dr. Rajewsky (Max Delbrück Center for Molecular Medicine)
Genetic reagent (*M. musculus*)	B1-8^hi^ki	Shih et al., 2002	IMSR Cat# JAX:007775; RRID:IMSR_JAX:007775	Dr. Nussenzweig (The Rockefeller University)
Genetic reagent (M. musculus)	*Bcl6*^flox^	Kaji et al., 2012	IMSR Cat# RBRC05663; RRID:IMSR_RBRC05663	Dr. Takemori (RIKEN)
Genetic reagent (*M. musculus*)	*Cd4*-Cre	Lee et al., 2001	IMSR Cat# JAX:017336; RRID:IMSR_JAX:017336	Dr. Kubo (Tokyo University of Science)
Genetic reagent (*M. musculus*)	*Cd40lg*^−/−^	Xu et al., 1994	RRID:MGI:2449454	Dr. Flavell (Yale School of Medicine)
Genetic reagent (*M. musculus*)	Igκ^−/−^	Chen et al., 1993		Dr. Tsubata (Tokyo Medical and Dental University)
Genetic reagent (*M. musculus*)	OT-II	Barnden et al., 1998	IMSR Cat# JAX:004194; RRID:IMSR_JAX:004194	Dr. Kubo (Tokyo University of Science)
Cell line (*M. musculus*)	40LB	Nojima et al., 2011		Dr. Kitamura (Tokyo University of Science)
Cell line (*M. musculus*)	40LB-hi	Takatsuka et al., 2018		Dr. Kitamura (Tokyo University of Science)
Cell line (*M. musculus*)	40LB-lo	this paper		Dr. Kitamura (Tokyo University of Science)
Cell line (*M. musculus*)	40LB-mid	this paper		Dr. Kitamura (Tokyo University of Science)
Antibody	rat monoclonal anti-mouse CD40; FGK4.5	Bio X Cell	Bio X Cell Cat# BE0016-2; RRID:AB_1107647	250 μg
Antibody	armenian hamster monoclonal anti-mouse CD40L; MR-1	Noelle et al., 1992	ATCC Cat# HB-11048; RRID:CVCL_8964	1 mg/kg; Dr. Abe (Tokyo University of Science)
Sequence-based reagent	shRNA	this paper		See Supplementary file 1
Software, algorithm	FlowJo	https://www.flowjo.com/solutions/flowjo	RRID:SCR_008520	
Software, algorithm	GraphPad Prism	https://graphpad.com	RRID:SCR_002798	

### Mice and immunization

C57BL/6 NCrSlc (B6) mice were purchased from Sankyo Labo Service. All of the following mice were backcrossed to B6 or congenic B6 CD45.1^+^ mouse strains: B1-8 ki (Lam et al., 1997), B1-8^hi^ ki (Shih et al., 2002), *Bcl6*^f/f^ (Kaji et al., 2012), *Cd4*-Cre (Lee et al., 2001), *Cd40lg*^−/−^ (Xu et al., 1994), Igκ^−/−^ (Chen et al., 1993), and OT-II (Barnden et al., 1998). Mice were immunized i.p. with 100 μg of NP_32_-CGG, or NP_14_-OVA where indicated, in alum. Sex-matched, 7-week-old or older mice were used for all experiments. All mice were bred and maintained under specific pathogen-free conditions, and all animal experiments were performed under protocols approved by the Animal Care and Use Committee of the Tokyo University of Science (Approval No.: S15021, S16019, S17004, S18018).

### Flow cytometry

For all the flow cytometry (FCM) analyses, single-cell suspensions were depleted of red blood cells (RBC) by ammonium chloride lysis, blocked with anti-CD16/32 (FcγRII/III) Ab (2.4G2), and then stained with the appropriate mAbs listed in Supplementary file 1, in PBS supplemented with 0.5% BSA, 2 mM EDTA, and 0.05% sodium azide. Stained cells were analyzed using FACSCalibur or FACSCantoII (BD Biosciences) instruments. The data were analyzed using Flowjo (Tree Star). Dead cells, detected by using propidium iodide or Fixable Viability Dye (eBioscience), were gated out in all FCM experiments. For intracellular staining, cells were fixed and permeabilized using a Foxp3 staining kit (eBioscience) before staining.

### Cell purification and culture

Naïve B cells were purified as described previously (Nojima et al., 2011). Naïve T cells were purified from OT-II mice as follows: RBC-depleted splenocytes were stained with fluorochrome-conjugated mAbs to CD4, CD25, CD44, and CD62L, and then naïve T cells (CD4^+^ CD25^−^ CD44^−^ CD62L^+^) were sorted using FACSAriaII or FACSAriaIII (BD Biosciences) instruments. GC B cells were purified from the mice immunized with NP-CGG in alum 7 days previously as follows. Cells from pooled spleens were stained with FITC-conjugated anti-GL7 and anti-FITC microbeads (Miltenyi Biotec), and GL7^+^ cells were enriched using a MACS system (Miltenyi Biotec). The enriched cells were stained with anti-CD19, anti-CD38 and anti-CD138, and then GC B cells (CD19^+^ CD38^−^ CD138^−^ GL7^+^) were sorted using FACSAriaII or FACSAriaIII.

B and T cells were cultured in 37°C/5% CO_2_ conditions in complete medium: RPMI-1640 medium (Wako) supplemented with 10% heat-inactivated fetal bovine serum, 1 mM sodium pyruvate, 50 μM 2-mercaptoethanol, 10 mM HEPES pH7.5, 100 U/ml penicillin and 100 μg/ml streptomycin (GIBCO). Naïve B cells (5 × 10^6^/ml) were cultured with anti-CD40 (1C10; Southern Biotech) or IKKβ inhibitor (BAY11-7082; Merck). Sorted T cell subsets (2.5 × 10^5^/ml) were cultured with PMA (20 ng/ml; Sigma) and ionomycin (1 μg/ml; Sigma) for 2 hr. To generate Th0 cells, naïve OT-II T cells (1 × 10^6^/ml) were cultured in six-well plates (Corning) coated with anti-CD3ε (8 μg/ml; 145–2 C11; Biolegend) and anti-CD28 (8 μg/ml; 37.51; Biolegend) for 3 days, and then cultured without Abs for 1 day. The resultant Th0 cells (1 × 10^6^/ml) and naïve B cells (1 × 10^6^/ml) were co-cultured with OVA peptide (Figure 4a). GC B cells (1 × 10^6^/ml) were cultured with anti-IgM (10 or 1 μg/ml; Jackson ImmunoResearch), anti-IgG (10 or 1 μg/ml; Jackson ImmunoResearch), anti-CD40 (1C10; 10 or 1 μg/ml), IL-4 (10 ng/ml; PeproTech), IL-21 (10 ng/ml; PeproTech) or BAFF (10 ng/ml) for 6 hr.

### Adoptive transfer and memory B cell purification

Naïve B cells were purified from B1-8ki CD45.1 mice and the frequency of NP^+^ cells was determined by FCM. The naïve B cells containing 1 × 10^4^ NP^+^ B cells per mouse were transferred into B6 mice, which were then immunized i.p. with NP-CGG in alum on the next day. Four weeks after the immunization, B_mem_ cells were purified from pooled spleens through two-step negative sorting and final positive sorting: cells stained with biotinylated antibodies against CD4, CD8a, CD11b, CD43, CD45.2, CD49b, and Ter119, followed by streptavidin particle DM (BD Biosciences), were negatively sorted sequentially with the iMag (BD) and MACS systems. The resultant cells were stained with fluorochrome-conjugated CD19, CD38, CD45.1 mAbs, and NP-BSA, and then B_mem_ cells (all positive) were sorted using FACSAriaII or FACSAriaIII.

### In vivo administration of antibodies

To inhibit CD40 signaling, mice were injected s.c. with the antagonistic CD40L mAb MR-1, (in house; 30 μg per mouse) or with control IgG (IR-AHT-GF, Innovative Research), every day from day −1 to day 5 after immunization. To activate CD40 signaling, mice were injected i.p. with agonistic CD40 mAb (FGK4.5, Bio X Cell; 250 μg per mouse) or PBS at day 8 after immunization.

### Cell lines, iGB cell culture and iMB cell generation

Production of cell lines, iGB cell culture and iMB cell generation were performed as previously described (Nojima et al., 2011). 40LB-hi cells were generated by repeated transduction of a CD40L expression vector (pMXs-CD40L-IRES-GFP) into 40LB cells (Takatsuka et al., 2018). 40LB-mid and 40LB-lo cells were subclones of 40LB cells made by cell sorting followed by limiting dilution. The CD40L expression level in 40LB-mid cells was equivalent to that of the parental 40LB cells. A parental cell line for 40LB, BALB/c 3T3 fibroblast (clone A31), was provided by RIKEN BRC, Japan. All the BALB/c 3T3-derived cell lines were checked routinely using the PCR Mycoplasma Detection Set (Takara) and proved to be mycoplasma-free.

### Adoptive transfer of iMB cells for immunization

Splenocytes from mice that had been transfected with iGB cells 2 week earlier were analyzed by FCM to estimate the numbers of donor-derived (CD45.1^+^) iMB cells. To examine the response of the iMB cells to a NP antigen in vivo, spleen B cells, including a fixed number of iMB cells derived from B1-8 ki B cells, were co-transferred with CGG-primed spleen cells into WT B6 mice, which were immunized i.p. with NP-CGG in alum on the next day.

### Plasmid constructions

BATF and IRF4 cDNAs were cloned by PCR using iGB cell mRNA. The ER^T2^ segment was fused to the 5′-terminus of the BATF or IRF4 cDNAs by ligations using PCR-generated de novo restriction enzyme sites. The BATF-HKE mutant (H55Q, K63D, and E77K) was generated by PCR-based mutagenesis (Iwata et al., 2017; Tussiwand et al., 2012). Constructs encoding BATF or BATF-HKE, each fused with ER^T2^, were cloned into a pMXs-IRES-GFP vector. A construct encoding IRF4 fused with ER^T2^ was cloned into a pMXs-IRES-CFP vector, derived from the pMXs-IRES-GFP, in which the GFP sequence was replaced with CFP. CA-IKKβ (S177E and S188E) (Mercurio et al., 1997), CA-Akt (E40K) (Arimura et al., 2004), and CA-MKK4 (S257E, T261D) constructs were cloned into the pMXs-IRES-GFP. For RNAi, the target sequences of shRNAs, as listed in Supplementary file 1, were inserted into a pSIREN-GFP vector, which was made by replacing a puromycin resistance gene in a pSIREN-RetroQ vector (Clontech) with an EGFP sequence.

### Retroviral transduction

Retroviral transduction of iGB cells was performed as previously described (Haniuda et al., 2016). For T cells, naïve T cells were stimulated with plate-coated anti-CD3ε (8 μg/mL) and anti-CD28 (8 μg/mL) for 36 hr, and then transduced with retrovirus vectors by spin-infection (Haniuda et al., 2016). Retroviral transduction of in-vivo-activated primary B cells and their transfer into mice were performed as previously described (Inoue et al., 2017). In brief, B1-8^hi^ ki mice were injected i.p. with NP-Ficoll (50 μg), and then B cells were purified from the spleens of these mice 6 hr later and stimulated in vitro with anti-CD40 Ab (2 μg/ml) for 18 hr. Cultured B cells were spin-infected with retroviral vectors and further cultured for 3 hr. The resultant viable B cells (1 × 10^6^) were transferred into WT mice for immunization with NP-CGG.

### Immunoblot analysis

Cells were lysed in cytoplasmic extraction (CE) buffer (10 mM HEPES pH 7.9, 10 mM KCl, 0.1 mM EDTA pH 8.0, 0.1 mM EGTA, and 1 mM DTT) for 10 min at 4°C and then NP-40 were added to the final concentration of 0.5%. The cell lysates were centrifuged and supernatants were collected as the cytosolic fraction. Precipitates were washed twice with CE buffer and the final precipitates were lysed in a nuclear extraction buffer (20 mM HEPES pH7.9, 400 mM NaCl, 1 mM EDTA pH 8.0, 1 mM EGTA, 25% glycerol, and 1 mM DTT) for 40 min at 4°C with aggressive mixing every 10 min. The lysates were centrifuged and the supernatants were used as the nuclear fractions. The cytosolic and nuclear fractions were mixed with SDS sample buffer, boiled, and used for SDS-PAGE, followed by immunoblotting using Abs listed in the Supplementary file 1.

### Quantitative RT-PCR

The procedures for RNA extraction and reverse transcription to cDNA have been described previously (Nojima et al., 2011). Quantitative real-time PCR was performed with a 7500 fast Real-time PCR system or with QuantStudio 3 (Applied Biosystems). Gene expression levels were determined by the relative standard curve method and normalized to that of *Gapdh*.

### ELISA

NP-specific IgG1 was detected by ELISA using NP-BSA as a plate-coated antigen as described previously (Nojima et al., 2011).

## Data Availability

All data generated or analysed during this study are included in the manuscript and supporting files. Source data files have been provided for Figures 1-7.

## Additional files

Supplementary file 1. The list of antibodies and reagents used in this study.

Transparent reporting form

## References

[bib1] Allen D, Simon T, Sablitzky F, Rajewsky K, Cumano A (1988). Antibody engineering for the analysis of affinity maturation of an anti-hapten response. The EMBO Journal.

[bib2] Anderson SM, Tomayko MM, Ahuja A, Haberman AM, Shlomchik MJ (2007). New markers for murine memory B cells that define mutated and unmutated subsets. The Journal of Experimental Medicine.

[bib3] Arimura Y, Shiroki F, Kuwahara S, Kato H, Dianzani U, Uchiyama T, Yagi J (2004). Akt is a neutral amplifier for th cell differentiation. Journal of Biological Chemistry.

[bib4] Arpin C, Banchereau J, Liu YJ (1997). Memory B cells are biased towards terminal differentiation: a strategy that may prevent repertoire freezing. The Journal of Experimental Medicine.

[bib5] Barnden MJ, Allison J, Heath WR, Carbone FR (1998). Defective TCR expression in transgenic mice constructed using cDNA-based alpha- and beta-chain genes under the control of heterologous regulatory elements. Immunology and Cell Biology.

[bib6] Berberich I, Shu GL, Clark EA (1994). Cross-linking CD40 on B cells rapidly activates nuclear factor-kappa B. Journal of Immunology.

[bib7] Breitfeld D, Ohl L, Kremmer E, Ellwart J, Sallusto F, Lipp M, Förster R (2000). Follicular B helper T cells express CXC chemokine receptor 5, localize to B cell follicles, and support immunoglobulin production. The Journal of Experimental Medicine.

[bib8] Chen J, Trounstine M, Kurahara C, Young F, Kuo CC, Xu Y, Loring JF, Alt FW, Huszar D (1993). B cell development in mice that lack one or both immunoglobulin kappa light chain genes. The EMBO Journal.

[bib9] Daniels MA, Teixeiro E (2015). TCR signaling in T cell memory. Frontiers in Immunology.

[bib10] Dogan I, Bertocci B, Vilmont V, Delbos F, Mégret J, Storck S, Reynaud CA, Weill JC (2009). Multiple layers of B cell memory with different effector functions. Nature Immunology.

[bib11] Engels N, König LM, Heemann C, Lutz J, Tsubata T, Griep S, Schrader V, Wienands J (2009). Recruitment of the cytoplasmic adaptor Grb2 to surface IgG and IgE provides antigen receptor-intrinsic costimulation to class-switched B cells. Nature Immunology.

[bib12] George AA, Sharma M, Singh BN, Sahoo NC, Rao KV (2006). Transcription regulation from a TATA and INR-less promoter: spatial segregation of promoter function. The EMBO Journal.

[bib13] Haniuda K, Fukao S, Kodama T, Hasegawa H, Kitamura D (2016). Autonomous membrane IgE signaling prevents IgE-memory formation. Nature Immunology.

[bib14] Hawkins ED, Turner ML, Wellard CJ, Zhou JH, Dowling MR, Hodgkin PD (2013). Quantal and graded stimulation of B lymphocytes as alternative strategies for regulating adaptive immune responses. Nature Communications.

[bib15] He JS, Subramaniam S, Narang V, Srinivasan K, Saunders SP, Carbajo D, Wen-Shan T, Hidayah Hamadee N, Lum J, Lee A, Chen J, Poidinger M, Zolezzi F, Lafaille JJ, Curotto de Lafaille MA (2017). IgG1 memory B cells keep the memory of IgE responses. Nature Communications.

[bib16] Heise N, De Silva NS, Silva K, Carette A, Simonetti G, Pasparakis M, Klein U (2014). Germinal center B cell maintenance and differentiation are controlled by distinct NF-κB transcription factor subunits. The Journal of Experimental Medicine.

[bib17] Inoue T, Shinnakasu R, Ise W, Kawai C, Egawa T, Kurosaki T (2017). The transcription factor Foxo1 controls germinal center B cell proliferation in response to T cell help. The Journal of Experimental Medicine.

[bib18] Ise W, Fujii K, Shiroguchi K, Ito A, Kometani K, Takeda K, Kawakami E, Yamashita K, Suzuki K, Okada T, Kurosaki T (2018). T follicular helper Cell-Germinal center B cell interaction strength regulates entry into plasma cell or recycling germinal center cell fate. Immunity.

[bib19] Iwata A, Durai V, Tussiwand R, Briseño CG, Wu X, Grajales-Reyes GE, Egawa T, Murphy TL, Murphy KM (2017). Quality of TCR signaling determined by differential affinities of enhancers for the composite BATF-IRF4 transcription factor complex. Nature Immunology.

[bib20] Jaiswal AI, Croft M (1997). CD40 ligand induction on T cell subsets by peptide-presenting B cells: implications for development of the primary T and B cell response. Journal of Immunology.

[bib21] Jost PJ, Ruland J (2017). Aberrant NF-kappaB signaling in lymphoma: mechanisms, consequences, and therapeutic implications. Blood.

[bib22] Kaisho T, Schwenk F, Rajewsky K (1997). The roles of gamma 1 heavy chain membrane expression and cytoplasmic tail in IgG1 responses. Science.

[bib23] Kaji T, Ishige A, Hikida M, Taka J, Hijikata A, Kubo M, Nagashima T, Takahashi Y, Kurosaki T, Okada M, Ohara O, Rajewsky K, Takemori T (2012). Distinct cellular pathways select germline-encoded and somatically mutated antibodies into immunological memory. The Journal of Experimental Medicine.

[bib24] Khalil AM, Cambier JC, Shlomchik MJ (2012). B cell receptor signal transduction in the GC is short-circuited by high phosphatase activity. Science.

[bib25] Knudson KM, Goplen NP, Cunningham CA, Daniels MA, Teixeiro E (2013). Low-affinity T cells are programmed to maintain normal primary responses but are impaired in their recall to low-affinity ligands. Cell Reports.

[bib26] Kometani K, Nakagawa R, Shinnakasu R, Kaji T, Rybouchkin A, Moriyama S, Furukawa K, Koseki H, Takemori T, Kurosaki T (2013). Repression of the transcription factor Bach2 contributes to predisposition of IgG1 memory B cells toward plasma cell differentiation. Immunity.

[bib27] Kräutler NJ, Suan D, Butt D, Bourne K, Hermes JR, Chan TD, Sundling C, Kaplan W, Schofield P, Jackson J, Basten A, Christ D, Brink R (2017). Differentiation of germinal center B cells into plasma cells is initiated by high-affinity antigen and completed by tfh cells. The Journal of Experimental Medicine.

[bib28] Laidlaw BJ, Schmidt TH, Green JA, Allen CD, Okada T, Cyster JG (2017). The Eph-related tyrosine kinase ligand Ephrin-B1 marks germinal center and memory precursor B cells. The Journal of Experimental Medicine.

[bib29] Lam KP, Kühn R, Rajewsky K (1997). In vivo ablation of surface immunoglobulin on mature B cells by inducible gene targeting results in rapid cell death. Cell.

[bib30] Lee PP, Fitzpatrick DR, Beard C, Jessup HK, Lehar S, Makar KW, Pérez-Melgosa M, Sweetser MT, Schlissel MS, Nguyen S, Cherry SR, Tsai JH, Tucker SM, Weaver WM, Kelso A, Jaenisch R, Wilson CB (2001). A critical role for Dnmt1 and DNA methylation in T cell development, function, and survival. Immunity.

[bib31] Lenschow DJ, Sperling AI, Cooke MP, Freeman G, Rhee L, Decker DC, Gray G, Nadler LM, Goodnow CC, Bluestone JA (1994). Differential up-regulation of the B7-1 and B7-2 costimulatory molecules after ig receptor engagement by antigen. Journal of Immunology.

[bib32] Liu W, Chen E, Zhao XW, Wan ZP, Gao YR, Davey A, Huang E, Zhang L, Crocetti J, Sandoval G, Joyce MG, Miceli C, Lukszo J, Aravind L, Swat W, Brzostowski J, Pierce SK (2012). The scaffolding protein synapse-associated protein 97 is required for enhanced signaling through isotype-switched IgG memory B cell receptors. Science Signaling.

[bib33] Luo W, Weisel F, Shlomchik MJ (2018). B cell receptor and CD40 signaling are rewired for synergistic induction of the c-Myc transcription factor in germinal center B cells. Immunity.

[bib34] Lutz J, Dittmann K, Bösl MR, Winkler TH, Wienands J, Engels N (2015). Reactivation of IgG-switched memory B cells by BCR-intrinsic signal amplification promotes IgG antibody production. Nature Communications.

[bib35] Mayer CT, Gazumyan A, Kara EE, Gitlin AD, Golijanin J, Viant C, Pai J, Oliveira TY, Wang Q, Escolano A, Medina-Ramirez M, Sanders RW, Nussenzweig MC (2017). The microanatomic segregation of selection by apoptosis in the germinal center. Science.

[bib36] McHeyzer-Williams LJ, Milpied PJ, Okitsu SL, McHeyzer-Williams MG (2015). Class-switched memory B cells remodel BCRs within secondary germinal centers. Nature Immunology.

[bib37] Mercurio F, Zhu H, Murray BW, Shevchenko A, Bennett BL, Li J, Young DB, Barbosa M, Mann M, Manning A, Rao A (1997). IKK-1 and IKK-2: cytokine-activated IkappaB kinases essential for NF-kappaB activation. Science.

[bib38] Mueller SN, Gebhardt T, Carbone FR, Heath WR (2013). Memory T cell subsets, migration patterns, and tissue residence. Annual Review of Immunology.

[bib39] Nishimura M, Murakami A, Hara Y, Azuma T (2011). Characterization of memory B cells responsible for affinity maturation of anti- (4-hydroxy-3-nitrophenyl)acetyl (NP) antibodies. International Immunology.

[bib40] Noelle RJ, Roy M, Shepherd DM, Stamenkovic I, Ledbetter JA, Aruffo A (1992). A 39-kDa protein on activated helper T cells binds CD40 and transduces the signal for cognate activation of B cells. PNAS.

[bib41] Nojima T, Haniuda K, Moutai T, Matsudaira M, Mizokawa S, Shiratori I, Azuma T, Kitamura D (2011). In-vitro derived germinal centre B cells differentially generate memory B or plasma cells in vivo. Nature Communications.

[bib42] Ochiai K, Maienschein-Cline M, Simonetti G, Chen J, Rosenthal R, Brink R, Chong AS, Klein U, Dinner AR, Singh H, Sciammas R (2013). Transcriptional regulation of germinal center B and plasma cell fates by dynamical control of IRF4. Immunity.

[bib43] Pape KA, Taylor JJ, Maul RW, Gearhart PJ, Jenkins MK (2011). Different B cell populations mediate early and late memory during an endogenous immune response. Science.

[bib44] Paus D, Phan TG, Chan TD, Gardam S, Basten A, Brink R (2006). Antigen recognition strength regulates the choice between extrafollicular plasma cell and germinal center B cell differentiation. The Journal of Experimental Medicine.

[bib45] Pepper M, Pagán AJ, Igyártó BZ, Taylor JJ, Jenkins MK (2011). Opposing signals from the Bcl6 transcription factor and the interleukin-2 receptor generate T helper 1 central and effector memory cells. Immunity.

[bib46] Phan TG, Paus D, Chan TD, Turner ML, Nutt SL, Basten A, Brink R (2006). High affinity germinal center B cells are actively selected into the plasma cell compartment. The Journal of Experimental Medicine.

[bib47] Randall TD, Heath AW, Santos-Argumedo L, Howard MC, Weissman IL, Lund FE (1998). Arrest of B lymphocyte terminal differentiation by CD40 signaling: mechanism for lack of antibody-secreting cells in germinal centers. Immunity.

[bib48] Ranheim EA, Kipps TJ (1993). Activated T cells induce expression of B7/BB1 on normal or leukemic B cells through a CD40-dependent signal. Journal of Experimental Medicine.

[bib49] Roy K, Mitchell S, Liu Y, Ohta S, Lin YS, Metzig MO, Nutt SL, Hoffmann A (2019). A regulatory circuit controlling the dynamics of nfκb cRel transitions B cells from proliferation to plasma cell differentiation. Immunity.

[bib50] Saito M, Gao J, Basso K, Kitagawa Y, Smith PM, Bhagat G, Pernis A, Pasqualucci L, Dalla-Favera R (2007). A signaling pathway mediating downregulation of BCL6 in germinal center B cells is blocked by BCL6 gene alterations in B cell lymphoma. Cancer Cell.

[bib51] Satpathy S, Shenoy GN, Kaw S, Vaidya T, Bal V, Rath S, George A (2010). Inhibition of terminal differentiation of B cells mediated by CD27 and CD40 involves signaling through JNK. The Journal of Immunology.

[bib52] Schwickert TA, Victora GD, Fooksman DR, Kamphorst AO, Mugnier MR, Gitlin AD, Dustin ML, Nussenzweig MC (2011). A dynamic T cell-limited checkpoint regulates affinity-dependent B cell entry into the germinal center. The Journal of Experimental Medicine.

[bib53] Sciammas R, Li Y, Warmflash A, Song Y, Dinner AR, Singh H (2011). An incoherent regulatory network architecture that orchestrates B cell diversification in response to antigen signaling. Molecular Systems Biology.

[bib54] Shih TA, Roederer M, Nussenzweig MC (2002). Role of antigen receptor affinity in T cell-independent antibody responses in vivo. Nature Immunology.

[bib55] Suan D, Kräutler NJ, Maag JLV, Butt D, Bourne K, Hermes JR, Avery DT, Young C, Statham A, Elliott M, Dinger ME, Basten A, Tangye SG, Brink R (2017). CCR6 defines memory B cell precursors in mouse and human germinal centers, revealing Light-Zone location and predominant low antigen affinity. Immunity.

[bib56] Suzuki S, Honma K, Matsuyama T, Suzuki K, Toriyama K, Akitoyo I, Yamamoto K, Suematsu T, Nakamura M, Yui K, Kumatori A (2004). Critical roles of interferon regulatory factor 4 in CD11bhighCD8alpha- dendritic cell development. PNAS.

[bib57] Takatsuka S, Yamada H, Haniuda K, Saruwatari H, Ichihashi M, Renauld JC, Kitamura D (2018). IL-9 receptor signaling in memory B cells regulates humoral recall responses. Nature Immunology.

[bib58] Tomayko MM, Steinel NC, Anderson SM, Shlomchik MJ (2010). Cutting edge: hierarchy of maturity of murine memory B cell subsets. The Journal of Immunology.

[bib59] Tussiwand R, Lee WL, Murphy TL, Mashayekhi M, Kc W, Albring JC, Satpathy AT, Rotondo JA, Edelson BT, Kretzer NM, Wu X, Weiss LA, Glasmacher E, Li P, Liao W, Behnke M, Lam SS, Aurthur CT, Leonard WJ, Singh H, Stallings CL, Sibley LD, Schreiber RD, Murphy KM (2012). Compensatory dendritic cell development mediated by BATF-IRF interactions. Nature.

[bib60] Wang Y, Shi J, Yan J, Xiao Z, Hou X, Lu P, Hou S, Mao T, Liu W, Ma Y, Zhang L, Yang X, Qi H (2017). Germinal-center development of memory B cells driven by IL-9 from follicular helper T cells. Nature Immunology.

[bib61] Weinstein JS, Herman EI, Lainez B, Licona-Limón P, Esplugues E, Flavell R, Craft J (2016). TFH cells progressively differentiate to regulate the germinal center response. Nature Immunology.

[bib62] Weisel FJ, Zuccarino-Catania GV, Chikina M, Shlomchik MJ (2016). A temporal switch in the germinal center determines differential output of memory B and plasma cells. Immunity.

[bib63] Xu J, Foy TM, Laman JD, Elliott EA, Dunn JJ, Waldschmidt TJ, Elsemore J, Noelle RJ, Flavell RA (1994). Mice deficient for the CD40 ligand. Immunity.

[bib64] Zotos D, Coquet JM, Zhang Y, Light A, D'Costa K, Kallies A, Corcoran LM, Godfrey DI, Toellner KM, Smyth MJ, Nutt SL, Tarlinton DM (2010). IL-21 regulates germinal center B cell differentiation and proliferation through a B cell-intrinsic mechanism. The Journal of Experimental Medicine.

[bib65] Zuccarino-Catania GV, Sadanand S, Weisel FJ, Tomayko MM, Meng H, Kleinstein SH, Good-Jacobson KL, Shlomchik MJ (2014). CD80 and PD-L2 define functionally distinct memory B cell subsets that are independent of antibody isotype. Nature Immunology.

